# Supplementary material for: Thermal dynamics and coalescence of Au144(SR)60 clusters from a machine-learned potential
Source: Nat Commun. 2025 Dec 18;17:971. doi: 10.1038/s41467-025-67700-w (PMC12847723; doi:10.1038/s41467-025-67700-w)
Supplement: Supplementary file 1 — Supplementary Information [file 41467_2025_67700_MOESM1_ESM.pdf]

Supplementary Information for:

Thermal dynamics and coalescence of $Au_{144}(SR)_{60}$ clusters from a machine-learned potential

Maryam Sabooni Asre Hazer*, Sami Malola, and Hannu Häkkinen*

E-mail: maryam.a.sabooni@jyu.fi; hannu.j.hakkinen@jyu.fi

Supplementary Figures

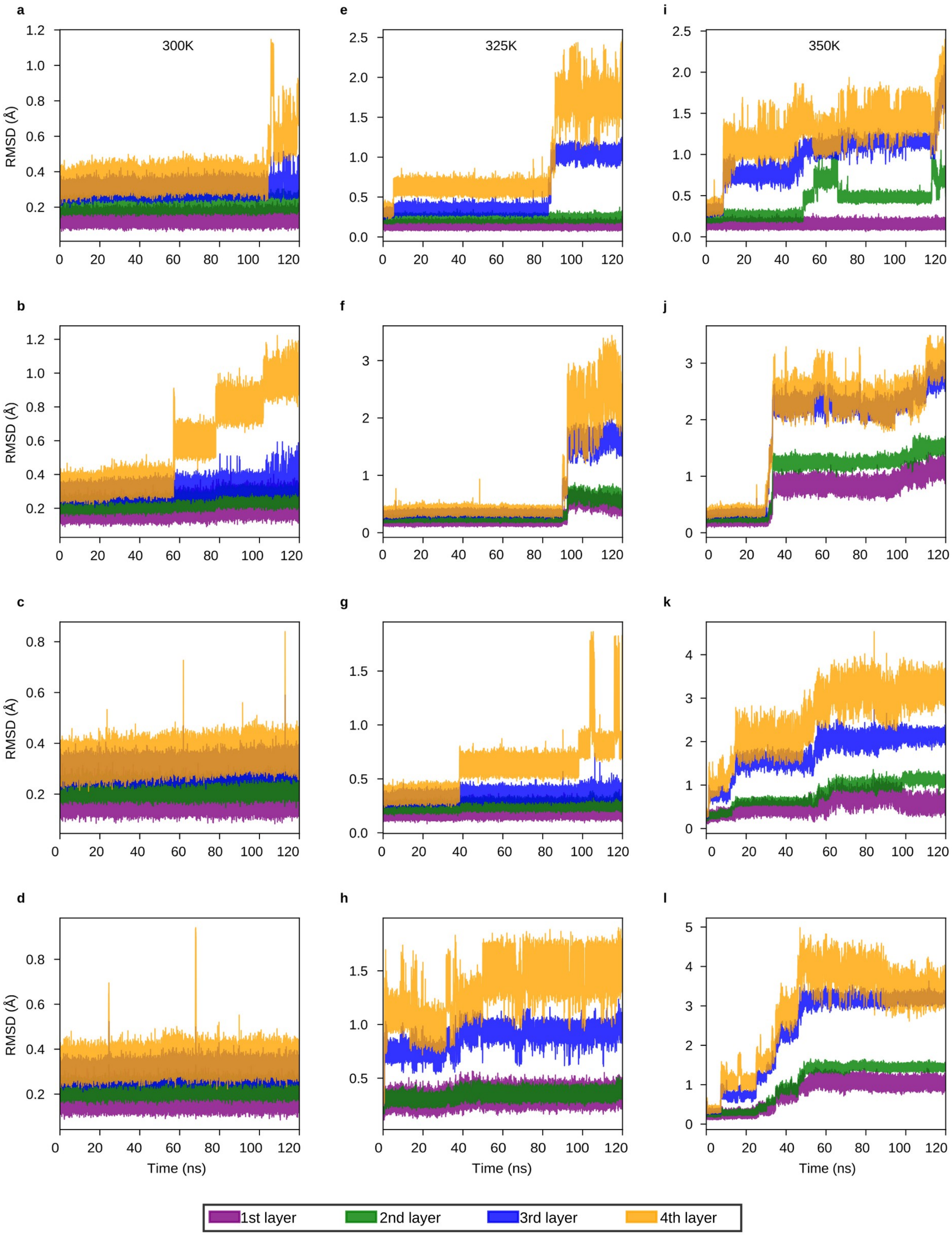

Supplementary Figure 1. Layer-resolved root mean square deviation (RMSD) of $Au_{144}(SCH_3)_{60}$ at 300-350 K. RMSD of $Au_{144}(SCH_3)_{60}$ for all Au atoms in four layers, as a function of simulation time (ns). Each layer represented by a different color: the 1st, 2nd, and 3rd layers are shown in purple, green, and blue, respectively, and the gold atoms in the ligand shell (referred to as the 4th layer) are depicted in orange. The RMSD analysis is performed for four replicas at 300 K (shown in a-d), 325 K (shown in e-h), and 350 K (shown in i-l).

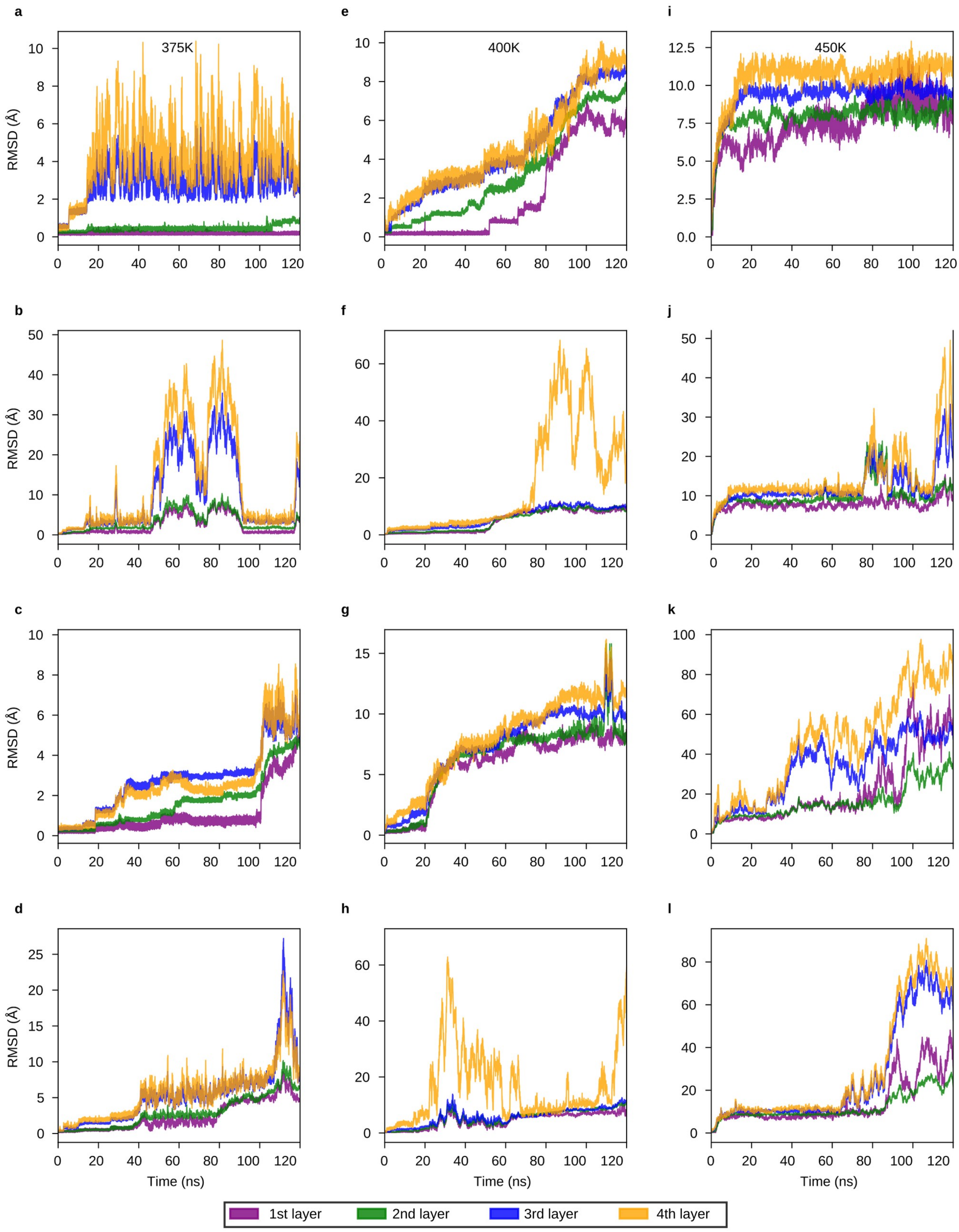

Supplementary Figure 2. Layer-resolved RMSD of $Au_{144}(SCH_3)_{60}$ at 375-450 K. RMSD of $Au_{144}(SCH_3)_{60}$ for all Au atoms in four layers, as a function of simulation time (ns). Each layer represented by a different color: the 1st, 2nd, and 3rd layers are shown in purple, green, and blue, respectively, and the gold atoms in the ligand shell (referred to as the 4th layer) are depicted in orange. The RMSD analysis is performed for four replicas at 375 K (shown in a-d), 400 K (shown in e-h), and 450 K (shown in i-l).

a

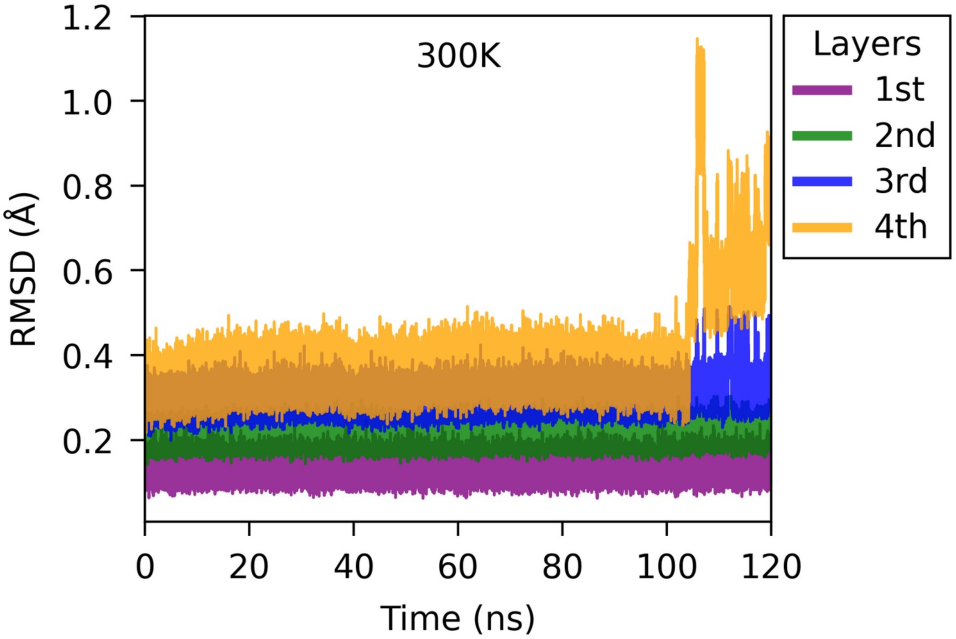

b

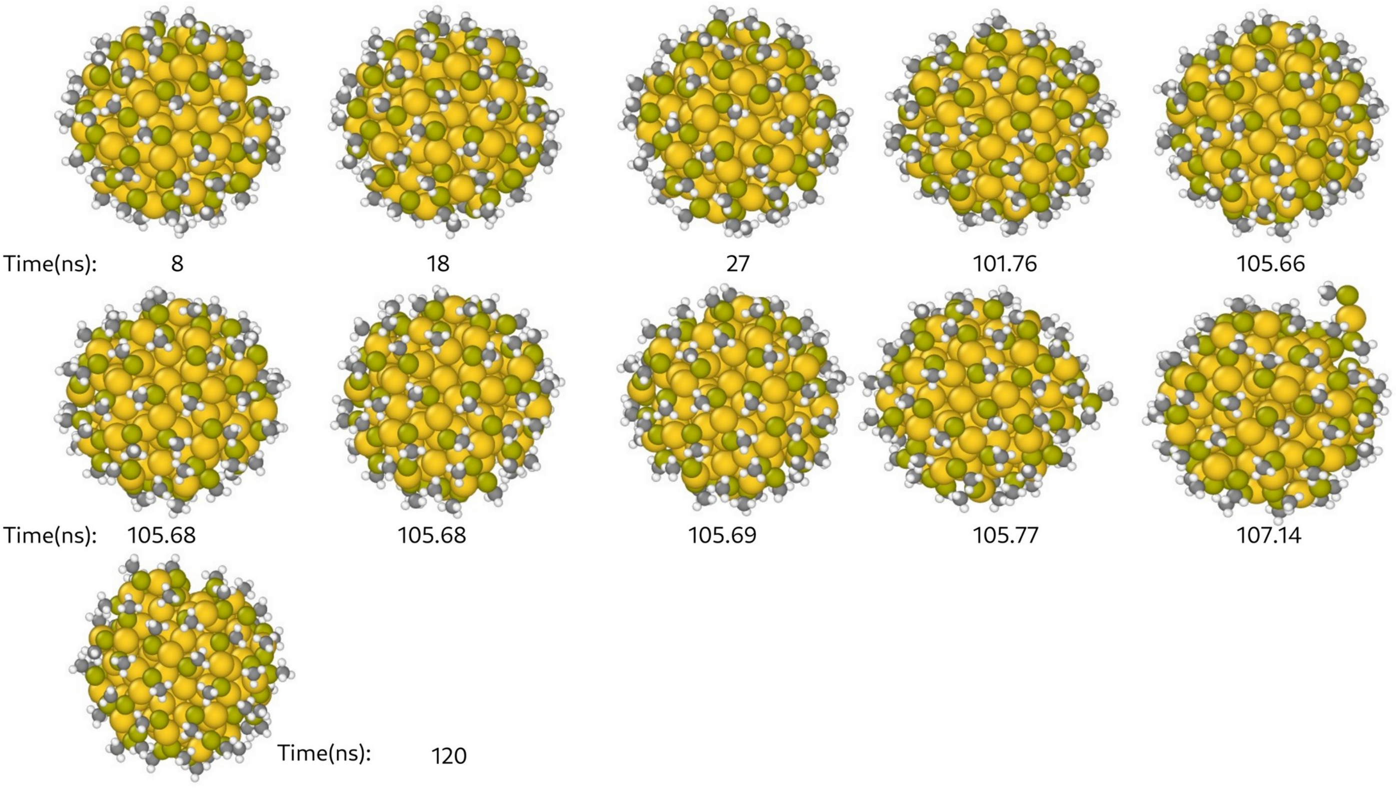

Supplementary Figure 3. Layer-resolved RMSD of $Au_{144}(SCH_3)_{60}$ at 300 K from the first replica (I). **a** RMSD of all atoms in $Au_{144}(SR)_{60}$ during MD simulation at **300 K (I)**. The RMSD plot tracks structural fluctuations over 120 ns. Layers are shown in purple (1st), green (2nd), blue (3rd), and orange (4th/in ligand shell). **b** Selected snapshots illustrating the atomic evolution at different time points (ns) during the MD simulation.

a

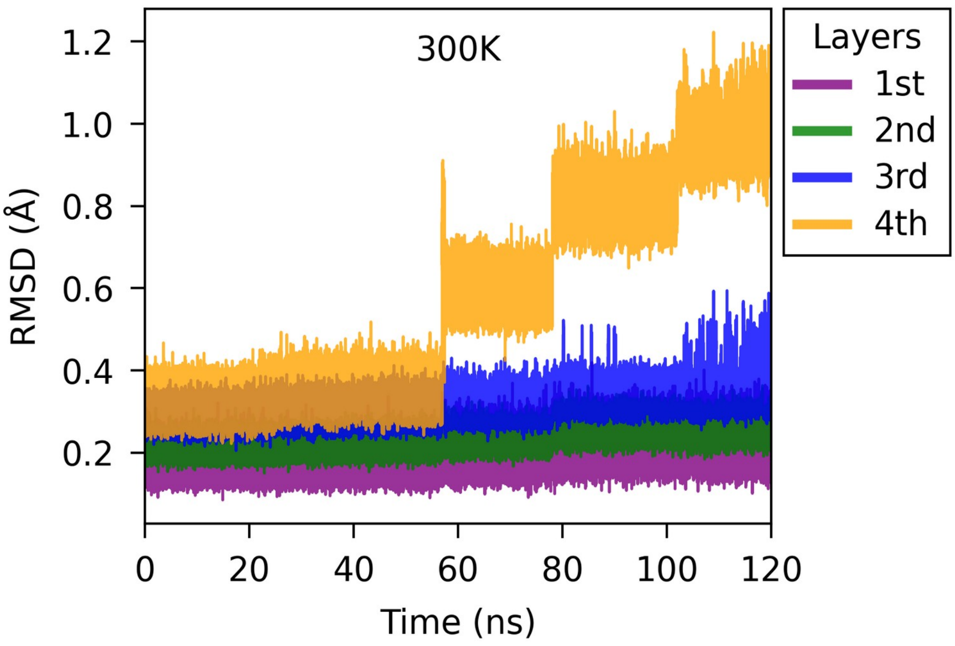

b

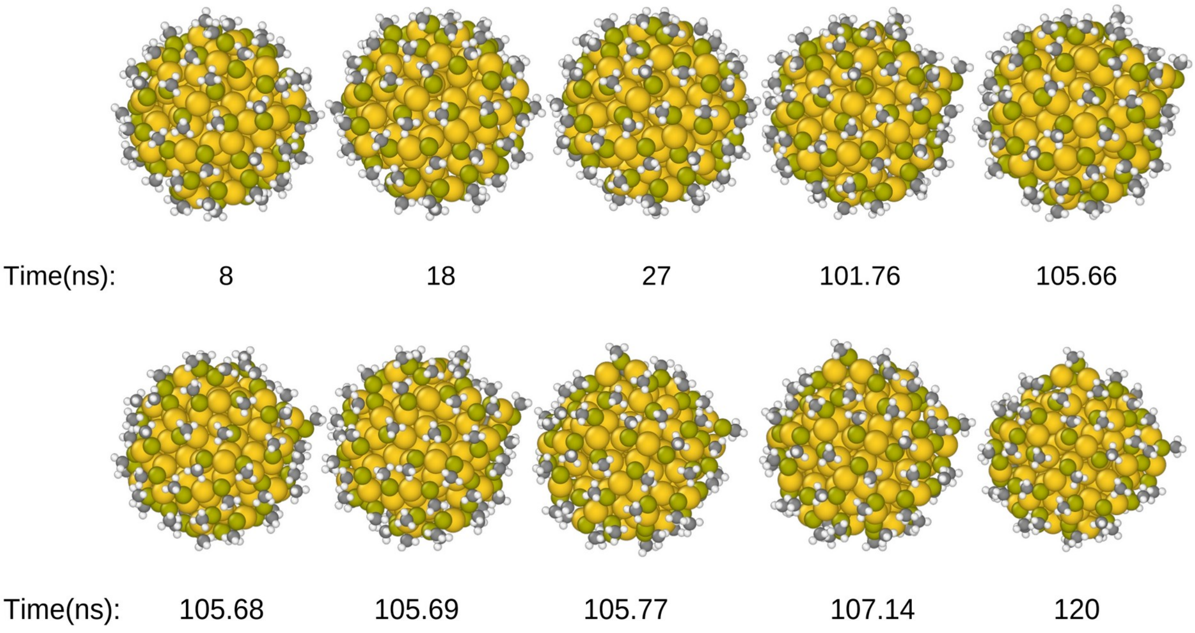

Supplementary Figure 4. Layer-resolved RMSD of $Au_{144}(SCH_3)_{60}$ at 300 K from the second replica (II). a RMSD of all atoms in $Au_{144}(SR)_{60}$ during MD simulation at **300 K (II)**. The RMSD plot tracks structural fluctuations over 120 ns. Layers are shown in purple (1st), green (2nd), blue (3rd), and orange (4th/in ligand shell). **b** Selected snapshots illustrating the atomic evolution at different time points (ns) during the MD simulation.

a

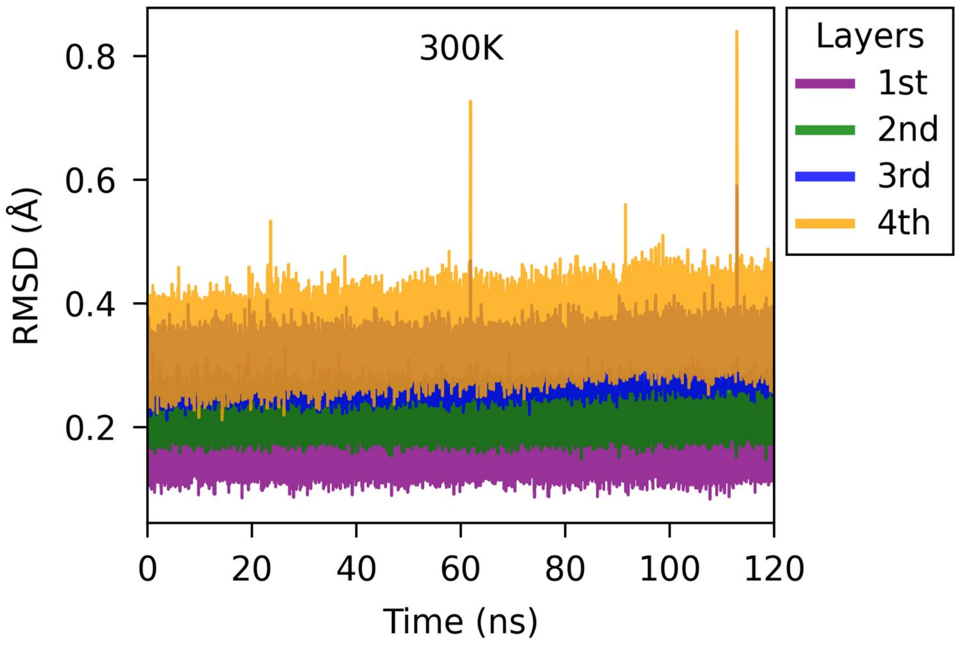

b

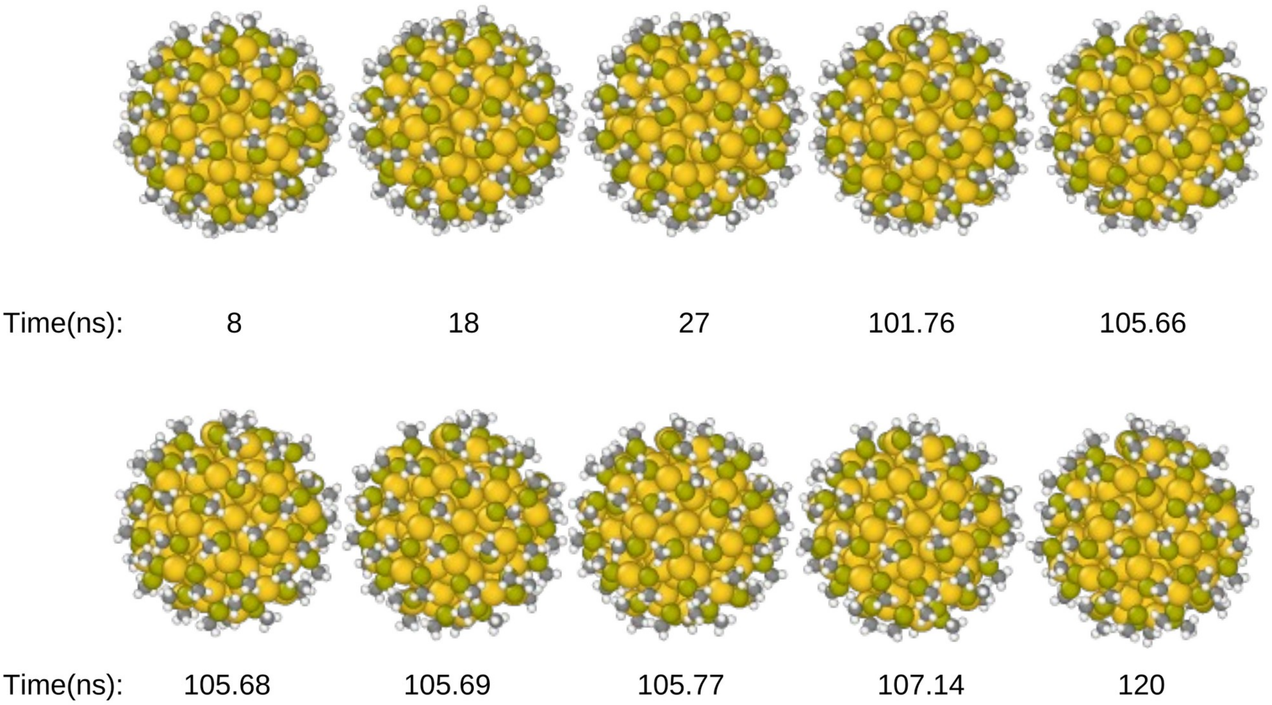

Supplementary Figure 5. Layer-resolved RMSD of $Au_{144}(SCH_3)_{60}$ at 300 K from the third replica (III). a RMSD of all atoms in $Au_{144}(SR)_{60}$ during MD simulation at **300 K (III)**. The RMSD plot tracks structural fluctuations over 120 ns. Layers are shown in purple (1st), green (2nd), blue (3rd), and orange (4th/in ligand shell). **b** Selected snapshots illustrating the atomic evolution at different time points (ns) during the MD simulation.

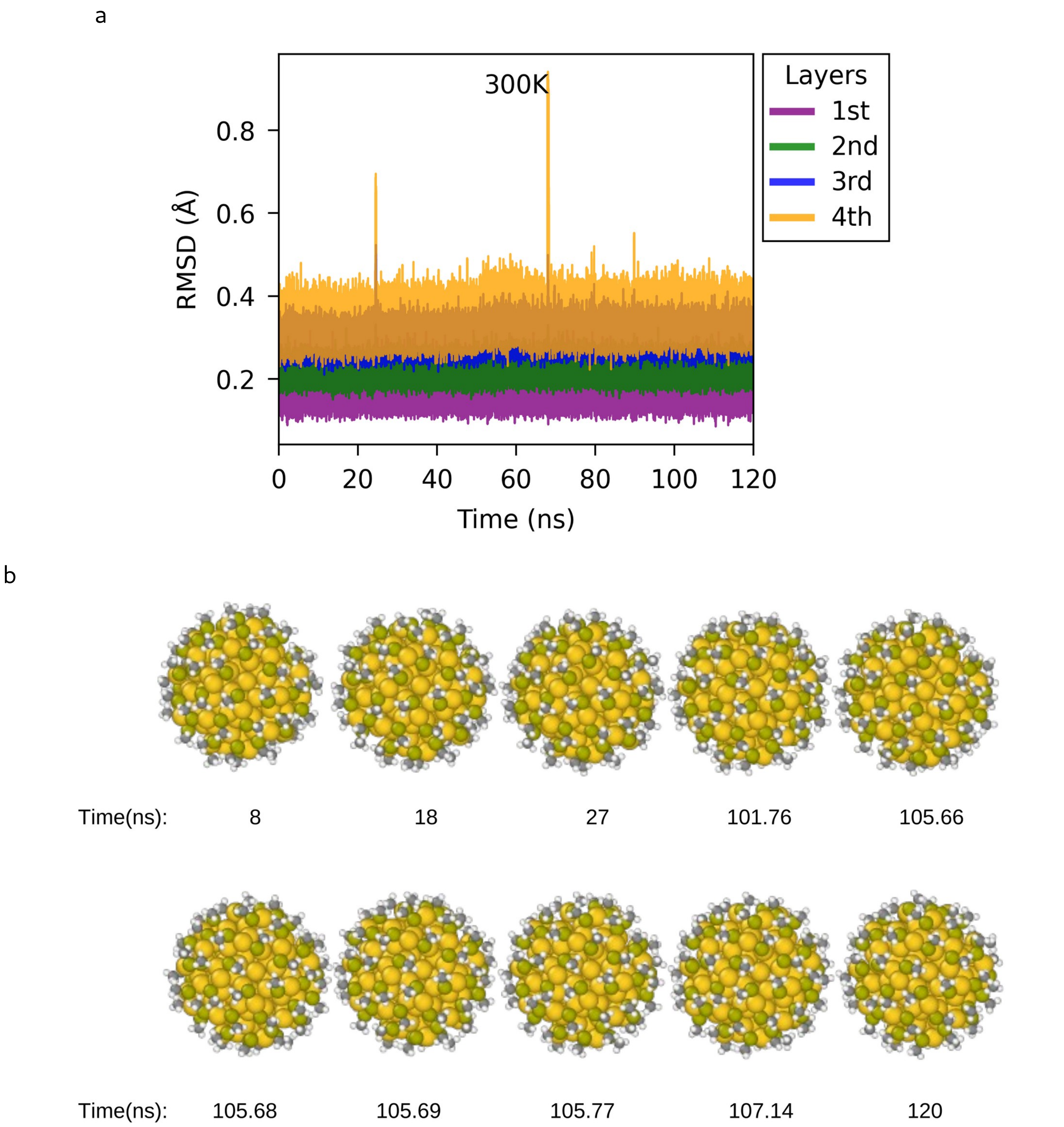

Supplementary Figure 6. Layer-resolved RMSD of $Au_{144}(SCH_3)_{60}$ at 300 K from the fourth replica (IV). a RMSD of all atoms in $Au_{144}(SR)_{60}$ during MD simulation at **300 K (IV)**. The RMSD plot tracks structural fluctuations over 120 ns. Layers are shown in purple (1st), green (2nd), blue (3rd), and orange (4th/in ligand shell). **b** Selected snapshots illustrating the atomic evolution at different time points (ns) during the MD simulation.

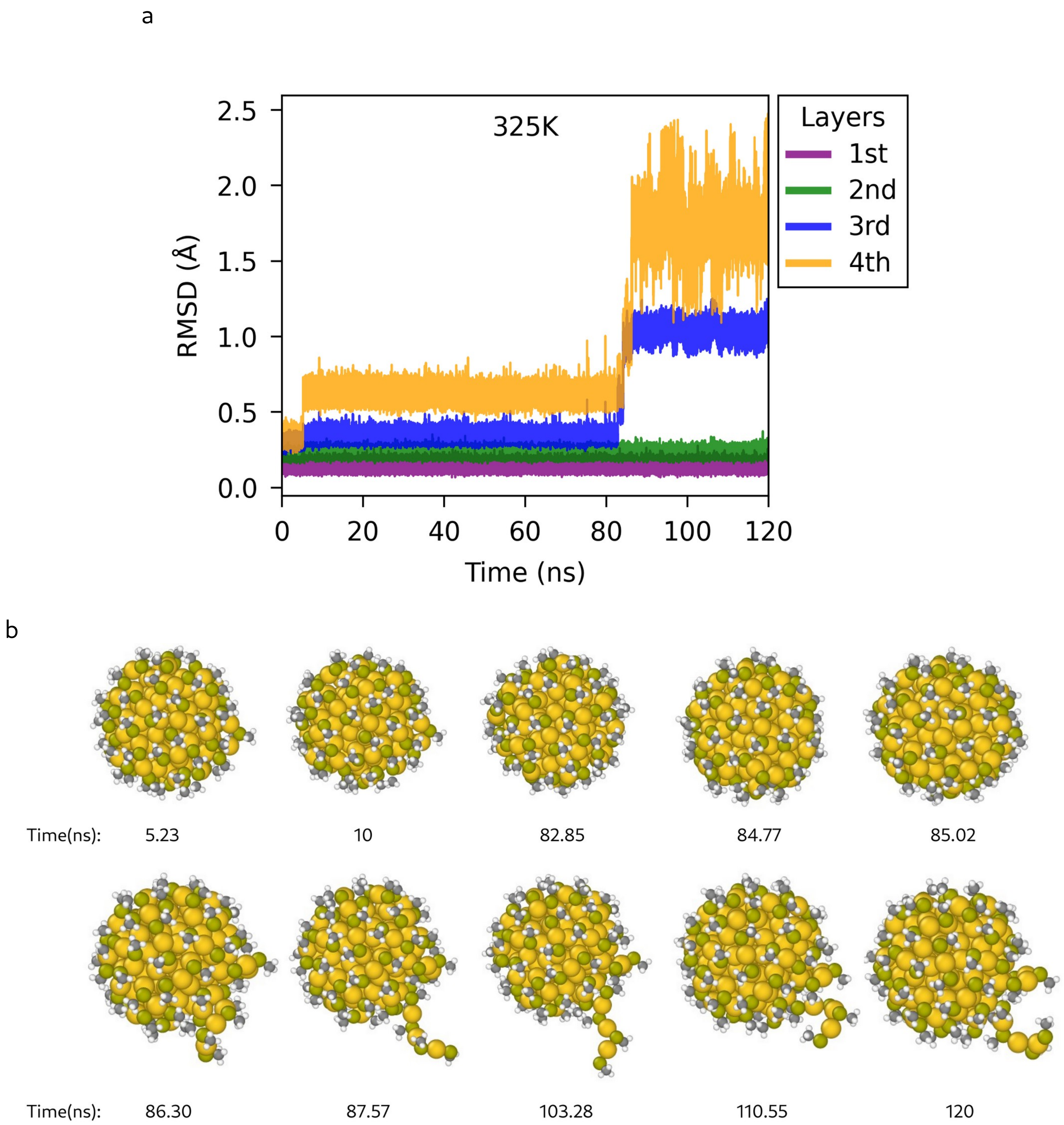

Supplementary Figure 7. Layer-resolved RMSD of $Au_{144}(SCH_3)_{60}$ at 325 K from the first replica (I). a RMSD of all atoms in $Au_{144}(SR)_{60}$ during MD simulation over 120 ns at **325 K (I)**. Layers are shown in purple (1st), green (2nd), blue (3rd), and orange (4th/in ligand shell). **b** with selected snapshots illustrating the atomic evolution at different time points (ns) during the MD simulation.

a

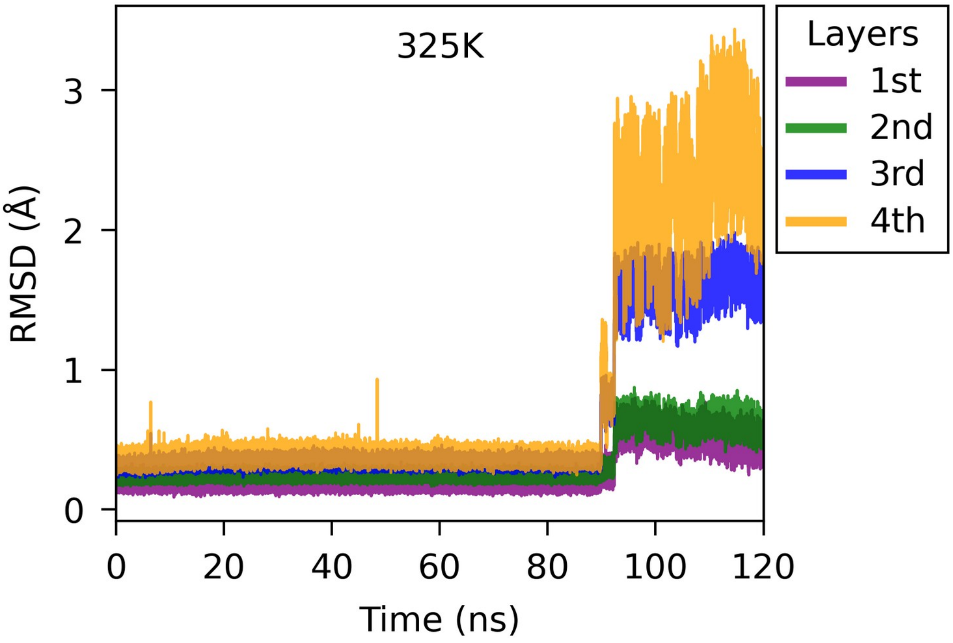

b

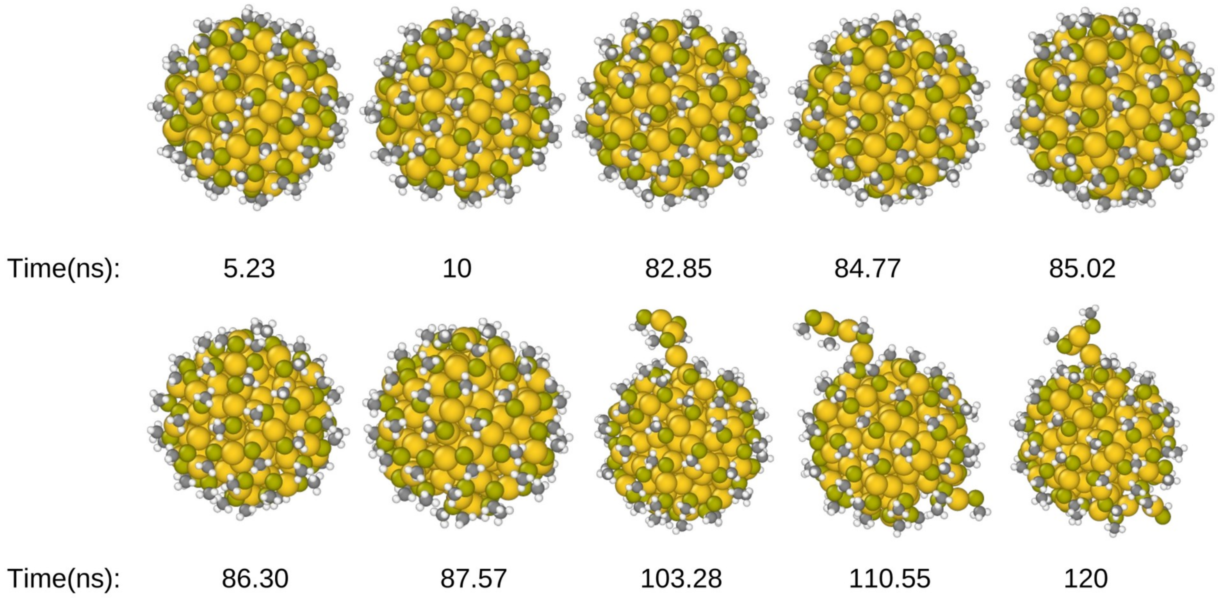

Supplementary Figure 8. Layer-resolved RMSD of $Au_{144}(SCH_3)_{60}$ at 325 K from the second replica (II). a RMSD of all atoms in $Au_{144}(SR)_{60}$ during MD simulation at **325 K (II)**. The RMSD plot tracks structural fluctuations over 120 ns. Layers are shown in purple (1st), green (2nd), blue (3rd), and orange (4th/in ligand shell). **b** Selected snapshots illustrating the atomic evolution at different time points (ns) during the MD simulation.

a

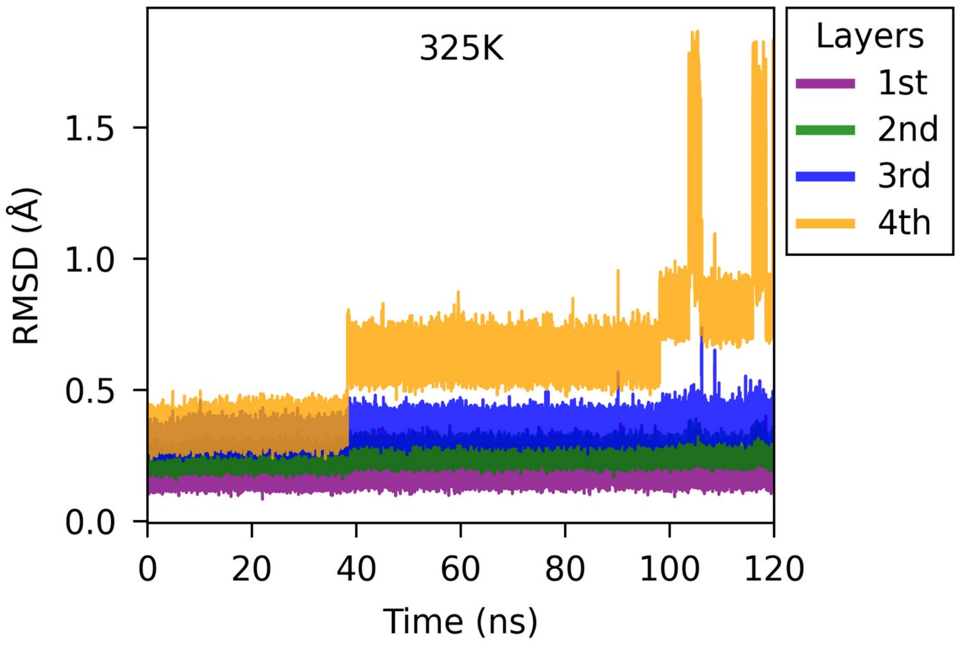

b

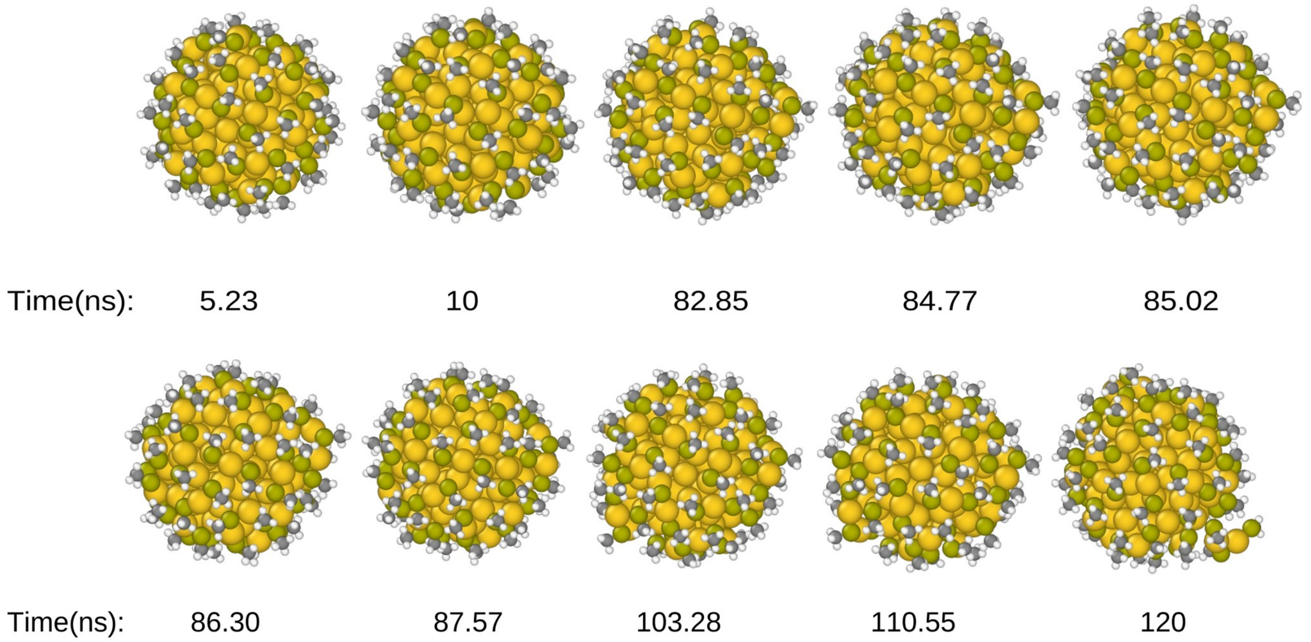

Supplementary Figure 9. Layer-resolved RMSD of $Au_{144}(SCH_3)_{60}$ at 325 K from the third replica (III). a RMSD of all atoms in $Au_{144}(SR)_{60}$ during MD simulation at **325 K (III)**. The RMSD plot tracks structural fluctuations over 120 ns. Layers are shown in purple (1st), green (2nd), blue (3rd), and orange (4th/in ligand shell). **b** Selected snapshots illustrating the atomic evolution at different time points (ns) during the MD simulation.

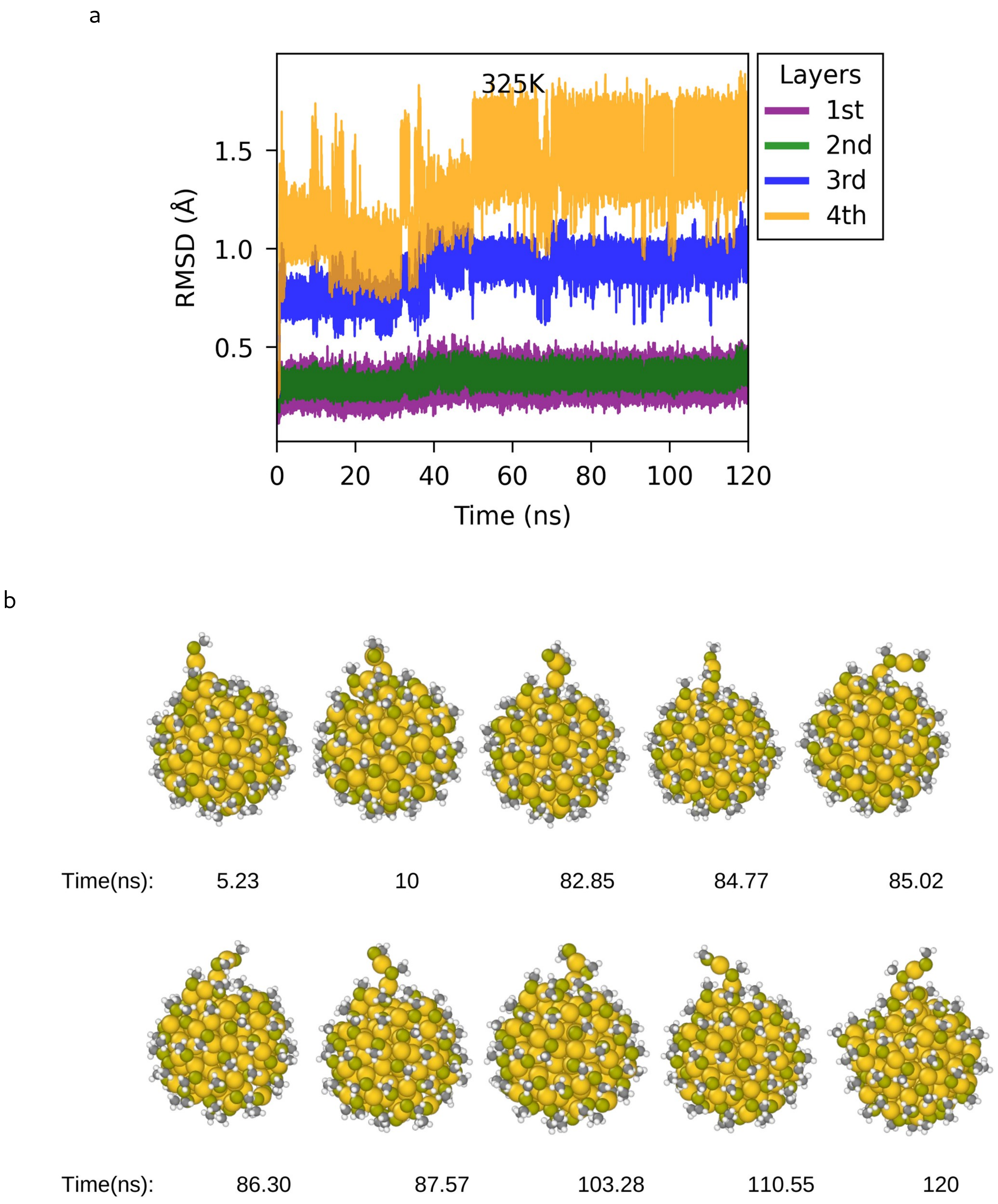

Supplementary Figure 10. Layer-resolved RMSD of $Au_{144}(SCH_3)_{60}$ at 325 K from the fourth replica (IV). a RMSD of all atoms in $Au_{144}(SR)_{60}$ during MD simulation at **325 K (IV)**. The RMSD plot tracks structural fluctuations over 120 ns. Layers are shown in purple (1st), green (2nd), blue (3rd), and orange (4th/in ligand shell). **b** Selected snapshots illustrating the atomic evolution at different time points (ns) during the MD simulation.

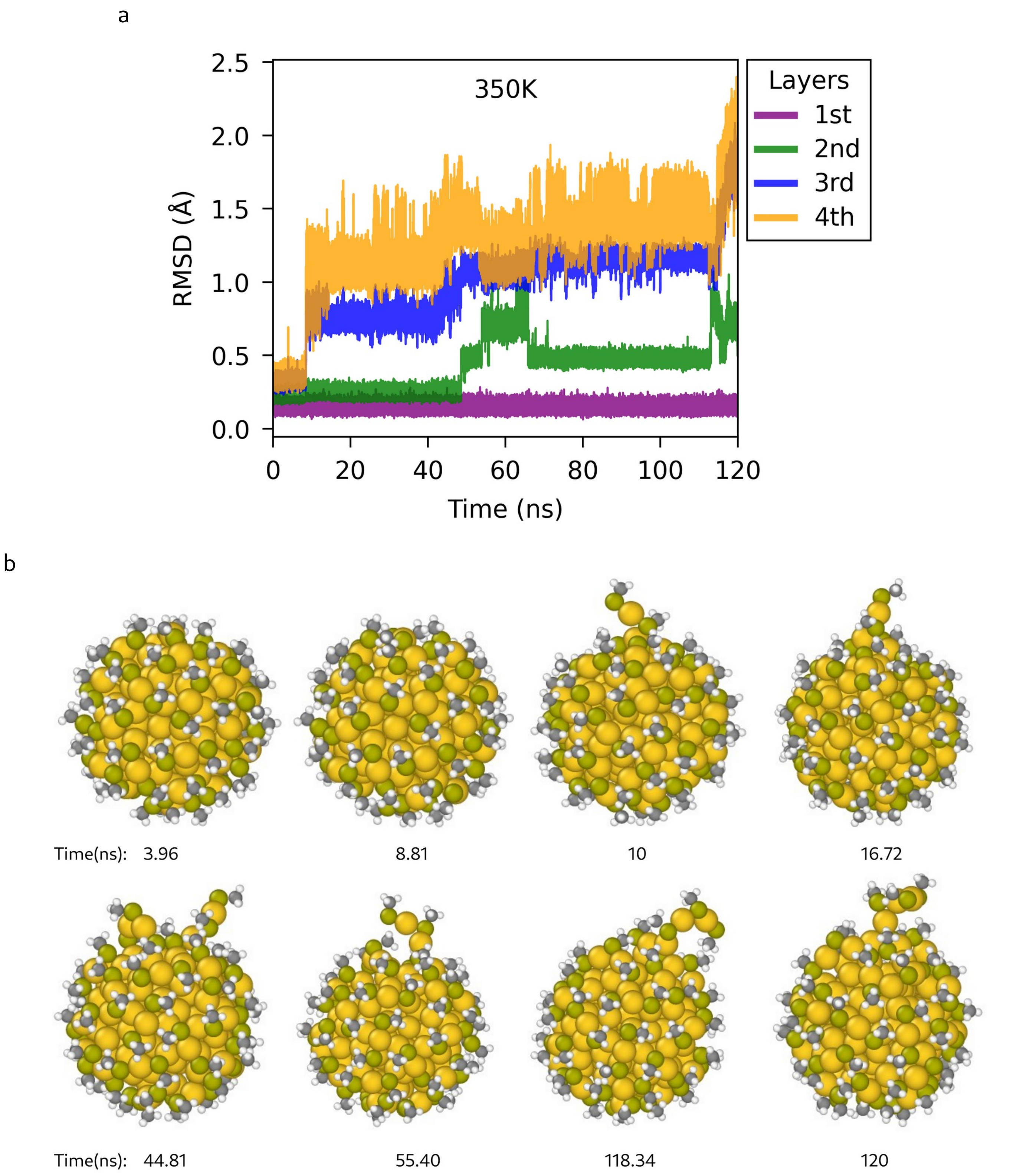

Supplementary Figure 11. Layer-resolved RMSD of $Au_{144}(SCH_3)_{60}$ at 350 K from the first replica (I). a RMSD of all atoms in $Au_{144}(SR)_{60}$ during MD simulation over 120 ns at **350 K (I)**. Layers are shown in purple (1st), green (2nd), blue (3rd), and orange (4th/in ligand shell). **b** Selected snapshots illustrating the atomic evolution at different time points (ns) during the MD simulation.

a

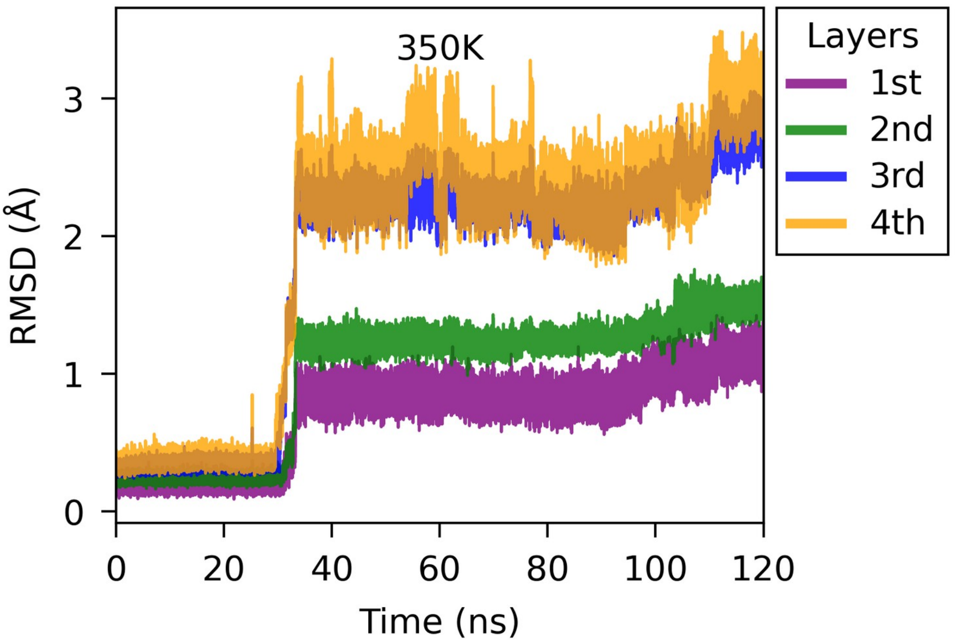

b

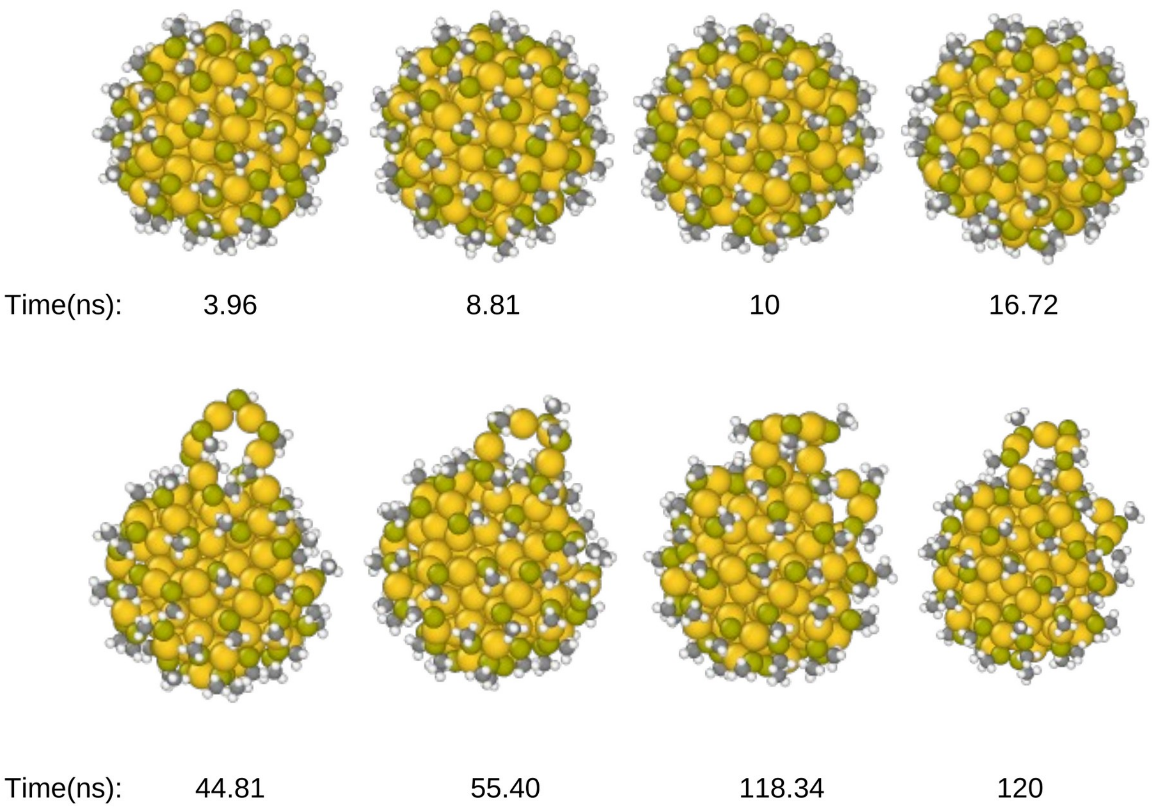

Supplementary Figure 12. Layer-resolved RMSD of $Au_{144}(SCH_3)_{60}$ at 350 K from the second replica (II). a RMSD of all atoms in $Au_{144}(SR)_{60}$ during MD simulation at **350 K (II)**. The RMSD plot tracks structural fluctuations over 120 ns. Layers are shown in purple (1st), green (2nd), blue (3rd), and orange (4th/in ligand shell). **b** Selected snapshots illustrating the atomic evolution at different time points (ns) during the MD simulation.

a

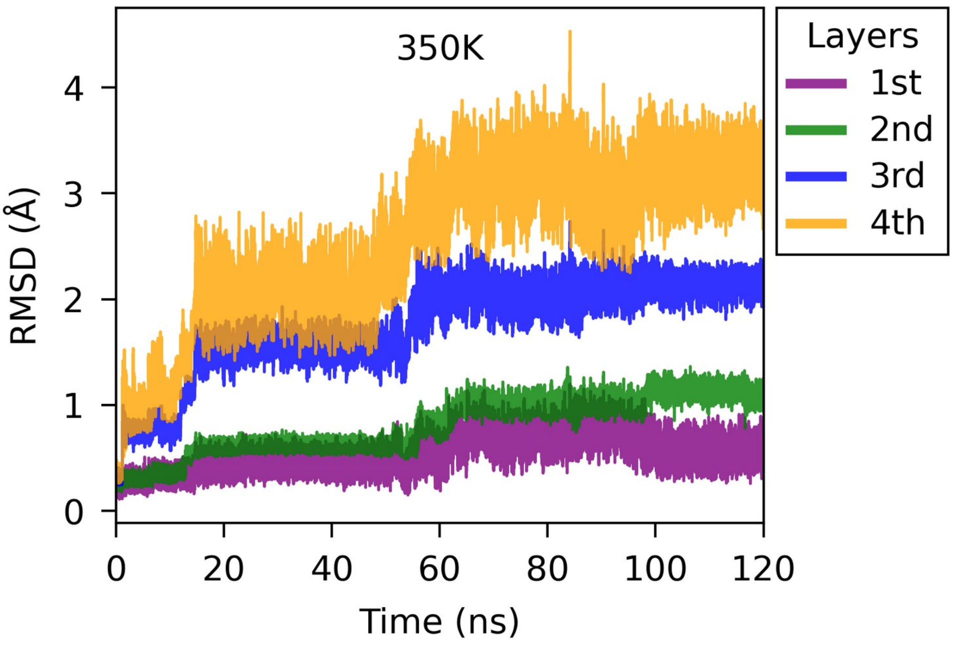

b

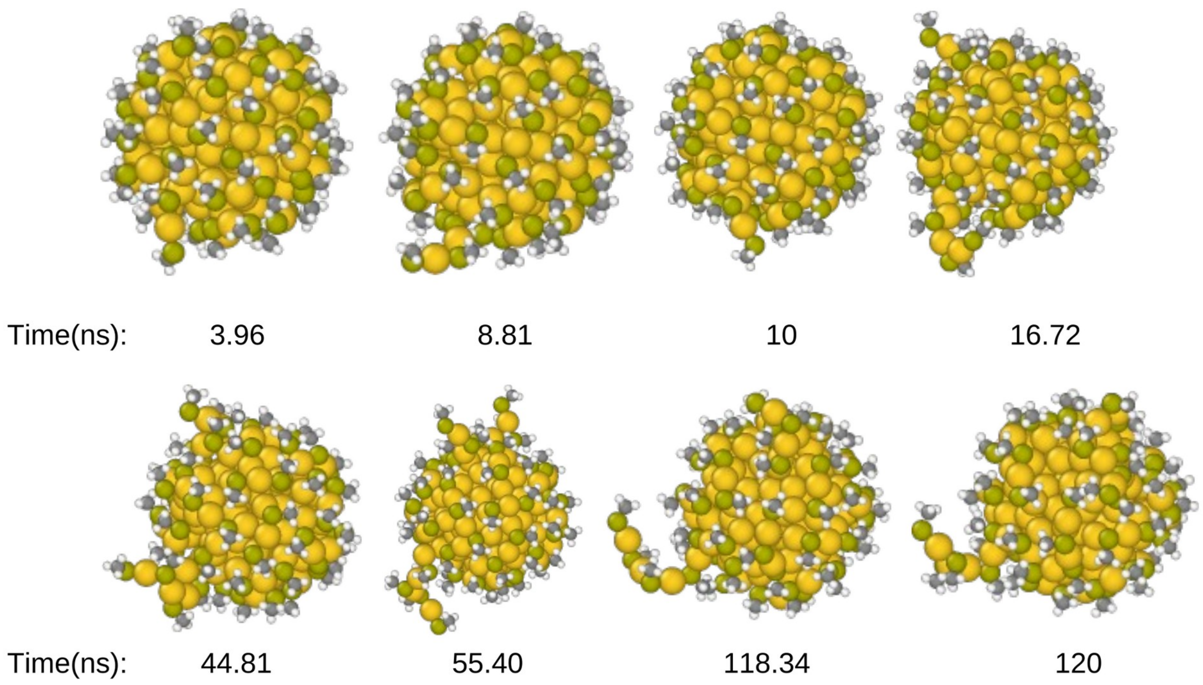

Supplementary Figure 13. Layer-resolved RMSD of $Au_{144}(SCH_3)_{60}$ at 350 K from the third replica (III). a RMSD of all atoms in $Au_{144}(SR)_{60}$ during MD simulation at **350 K (III)**. The RMSD plot tracks structural fluctuations over 120 ns. Layers are shown in purple (1st), green (2nd), blue (3rd), and orange (4th/in ligand shell). **b** Selected snapshots illustrating the atomic evolution at different time points (ns) during the MD simulation.

a

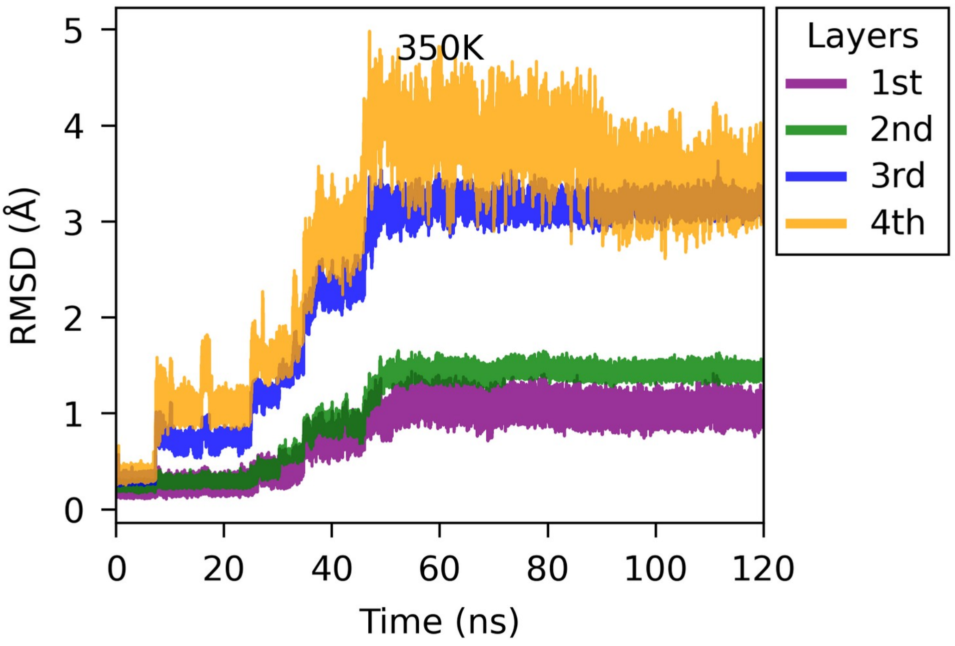

b

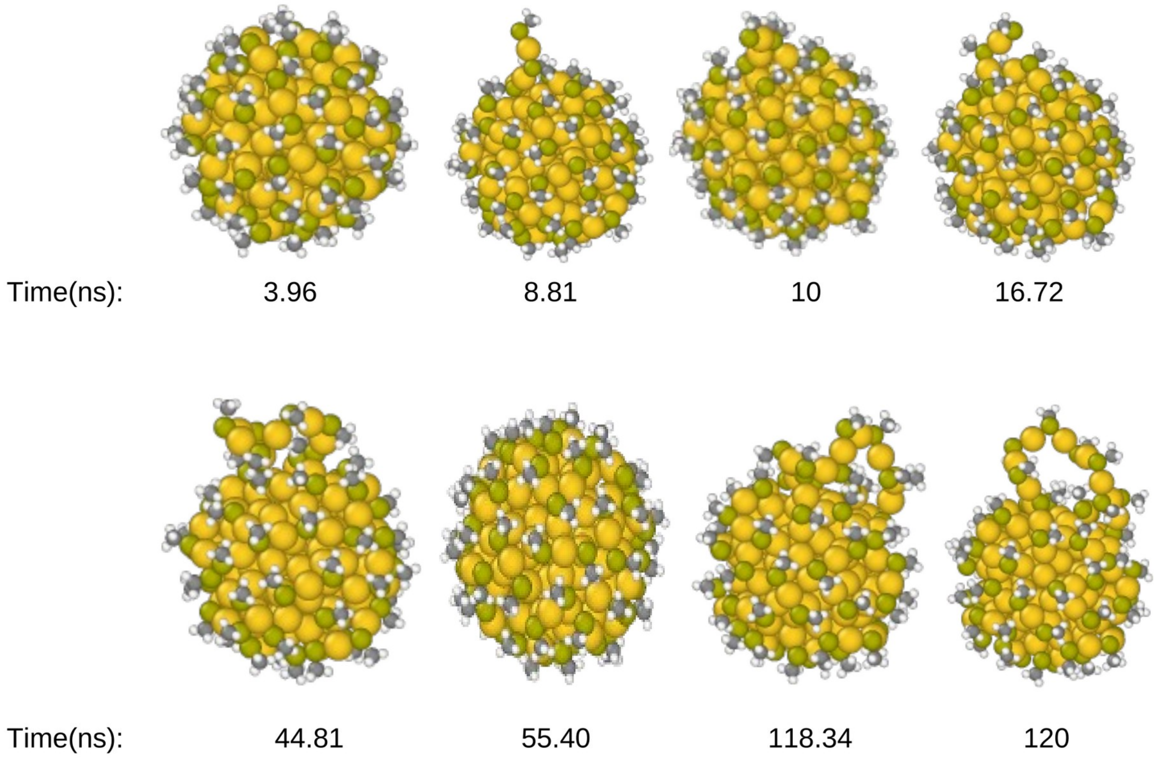

Supplementary Figure 14. Layer-resolved RMSD of $Au_{144}(SCH_3)_{60}$ at 350 K from the fourth replica (IV). a RMSD of all atoms in $Au_{144}(SR)_{60}$ during MD simulation at **350 K (IV)**. The RMSD plot tracks structural fluctuations over 120 ns. Layers are shown in purple (1st), green (2nd), blue (3rd), and orange (4th/in ligand shell). **b** Selected snapshots illustrating the atomic evolution at different time points (ns) during the MD simulation.

a

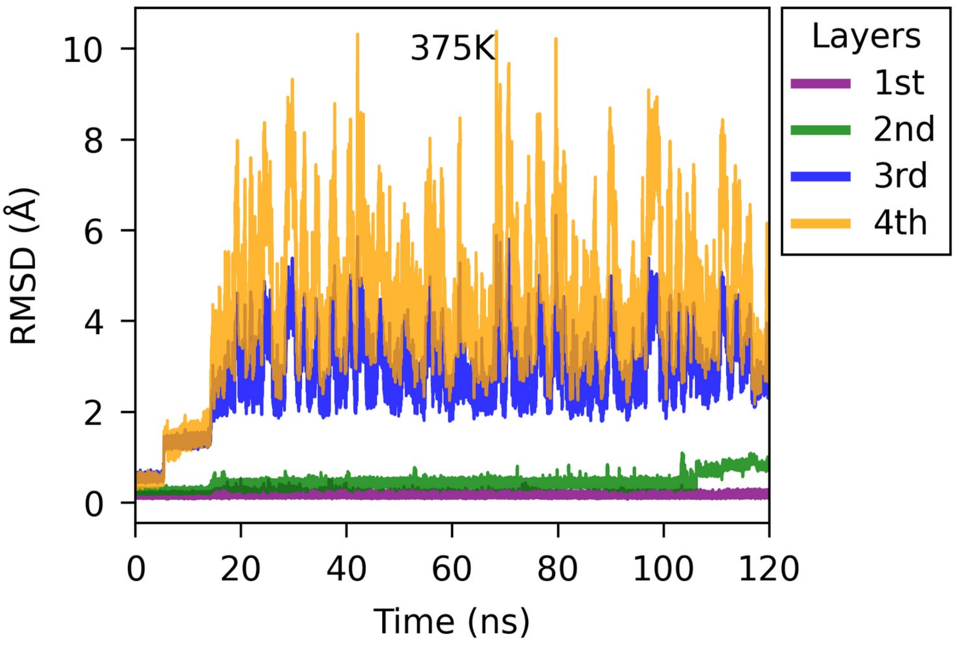

b

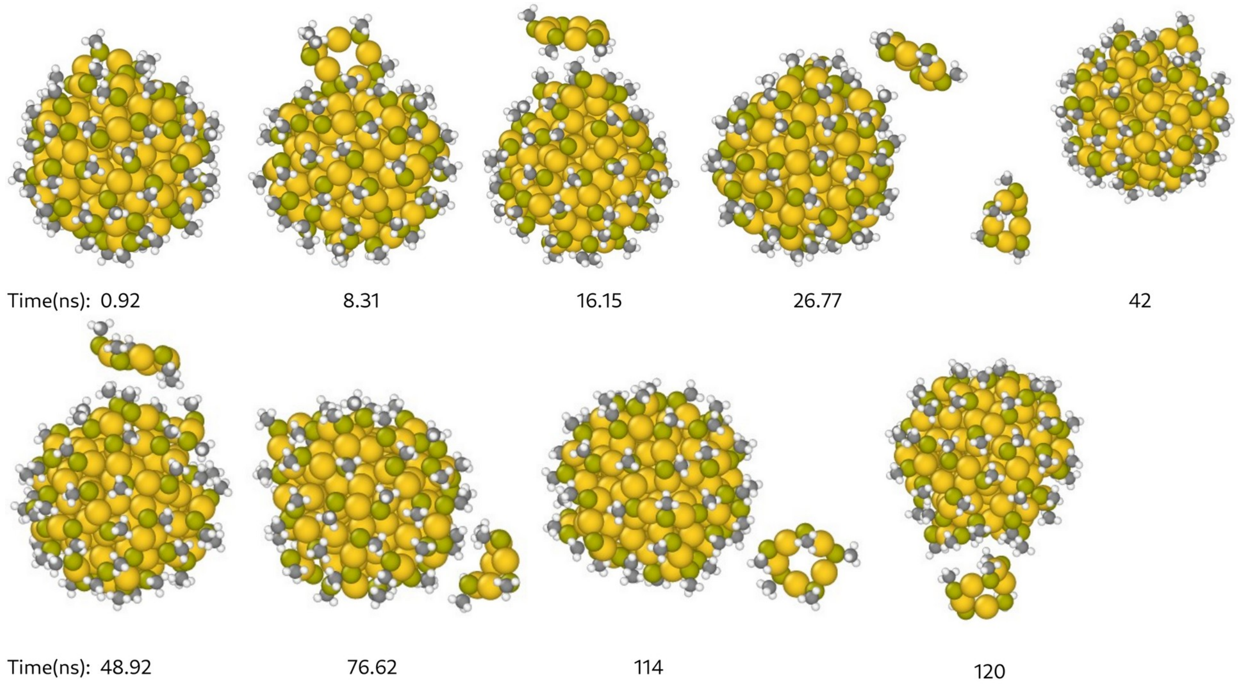

Supplementary Figure 15. Layer-resolved RMSD of $Au_{144}(SCH_3)_{60}$ at 375 K from the first replica (I). a RMSD of all atoms in $Au_{144}(SR)_{60}$ during MD simulation over 120 ns at **375 K (I)**. Layers are shown in purple (1st), green (2nd), blue (3rd), and orange (4th/in ligand shell). **b** Selected snapshots illustrating the atomic evolution at different time points (ns) during the MD simulation.

a

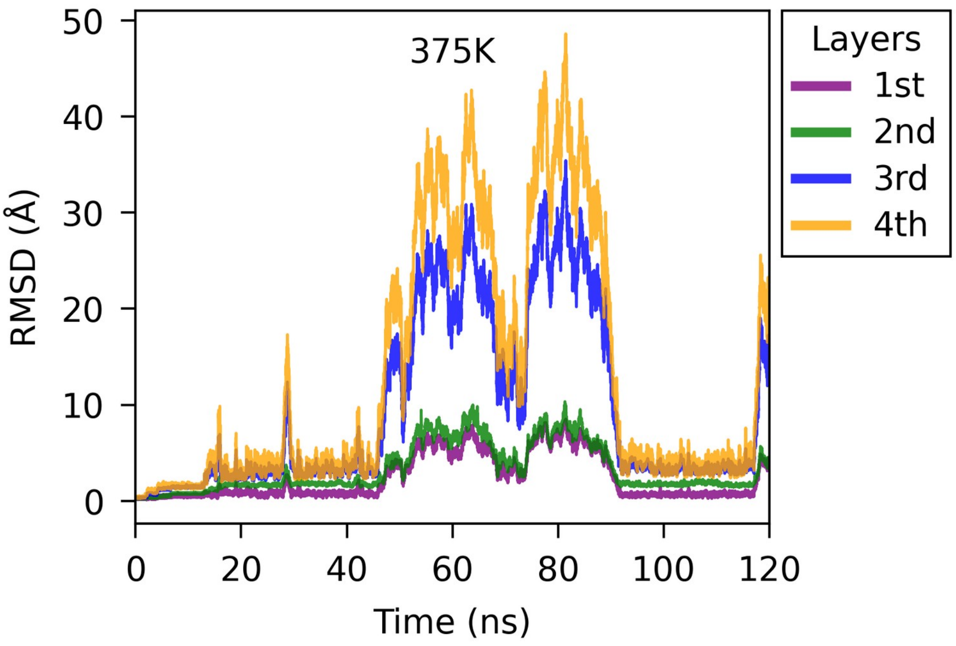

b

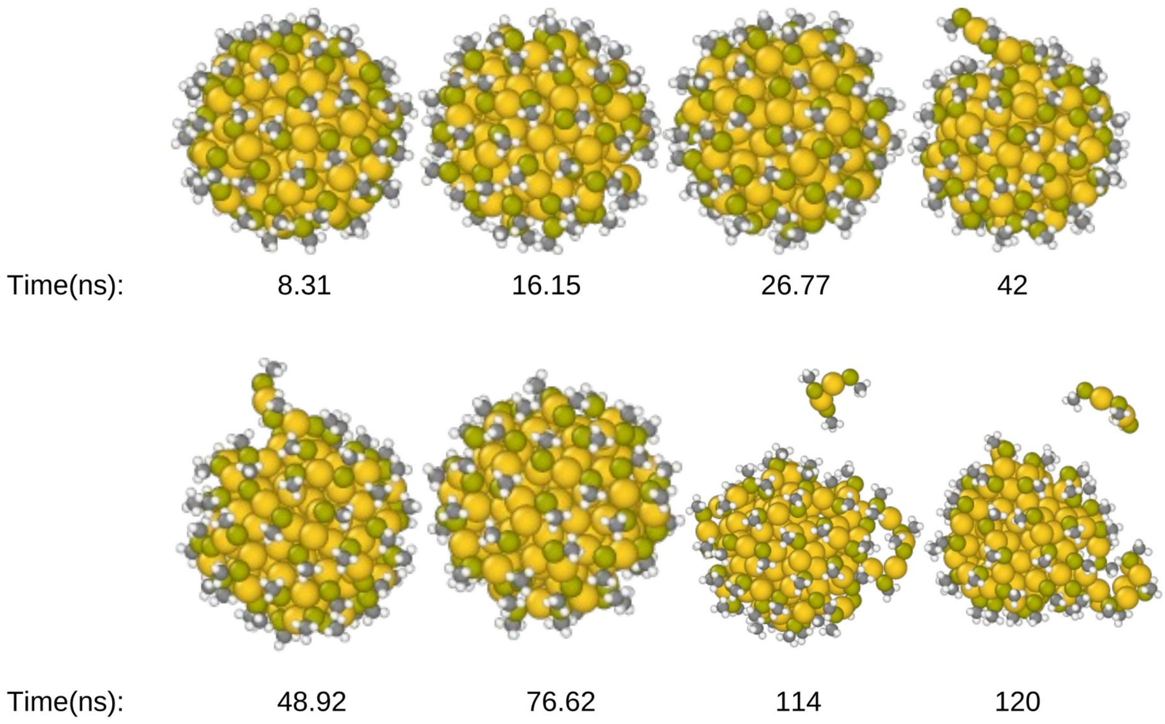

Supplementary Figure 16. Layer-resolved RMSD of $Au_{144}(SCH_3)_{60}$ at 375 K from the second replica (II). a RMSD of all atoms in $Au_{144}(SR)_{60}$ during MD simulation at **375 K (II)**. The RMSD plot tracks structural fluctuations over 120 ns. Layers are shown in purple (1st), green (2nd), blue (3rd), and orange (4th/in ligand shell). **b** Selected snapshots illustrating the atomic evolution at different time points (ns) during the MD simulation.

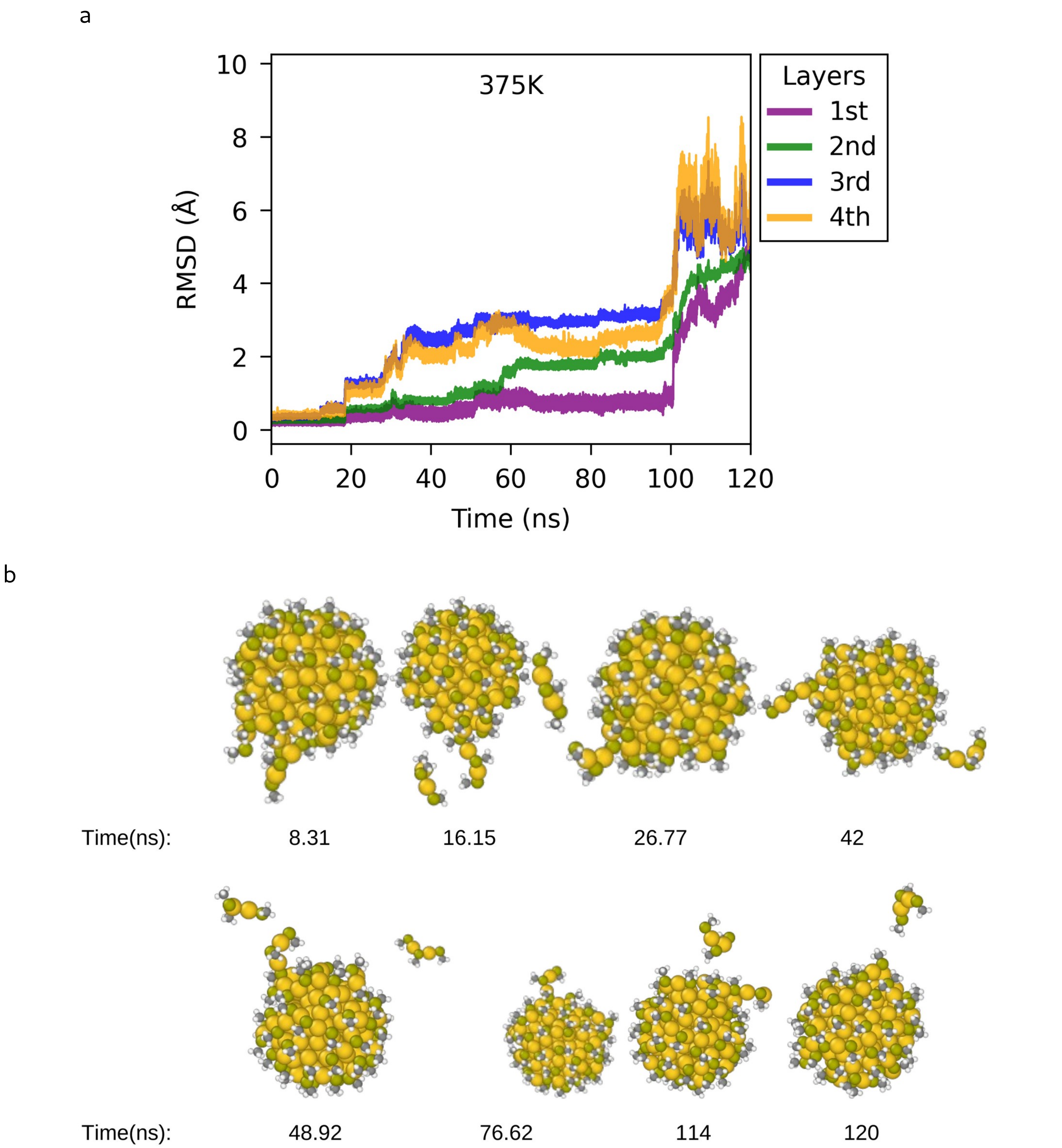

Supplementary Figure 17. Layer-resolved RMSD of $Au_{144}(SCH_3)_{60}$ at 375 K from the third replica (III). a RMSD of all atoms in $Au_{144}(SR)_{60}$ during MD simulation at **375 K (III)**. The RMSD plot tracks structural fluctuations over 120 ns. Layers are shown in purple (1st), green (2nd), blue (3rd), and orange (4th/in ligand shell). **b** Selected snapshots illustrating the atomic evolution at different time points (ns) during the MD simulation.

a

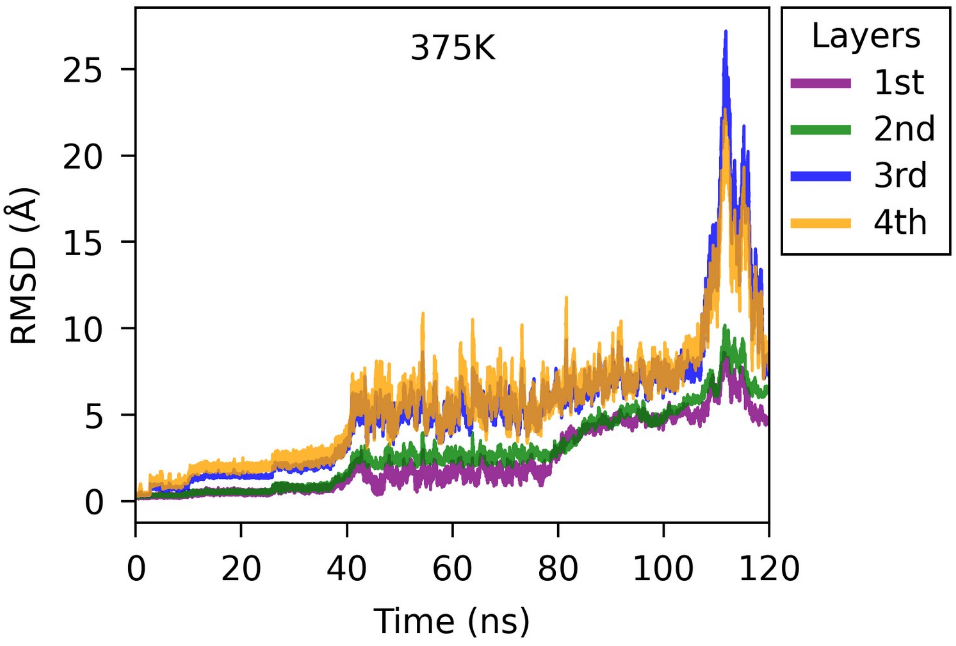

b

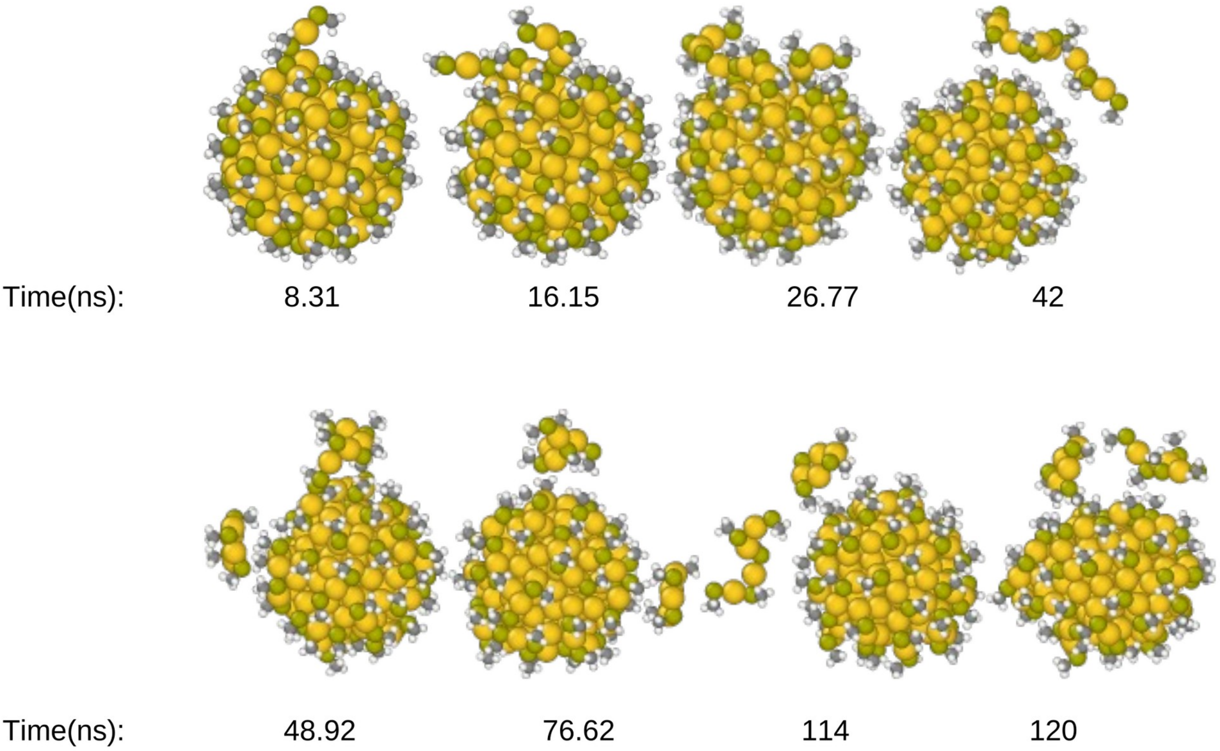

Supplementary Figure 18. Layer-resolved RMSD of $Au_{144}(SCH_3)_{60}$ at 375 K from the fourth MD replica (IV). a RMSD of all atoms in $Au_{144}(SR)_{60}$ during MD simulation at **375 K (IV)**. The RMSD plot tracks structural fluctuations over 120 ns. Layers are shown in purple (1st), green (2nd), blue (3rd), and orange (4th/in ligand shell). **b** Selected snapshots illustrating the atomic evolution at different time points (ns) during the MD simulation.

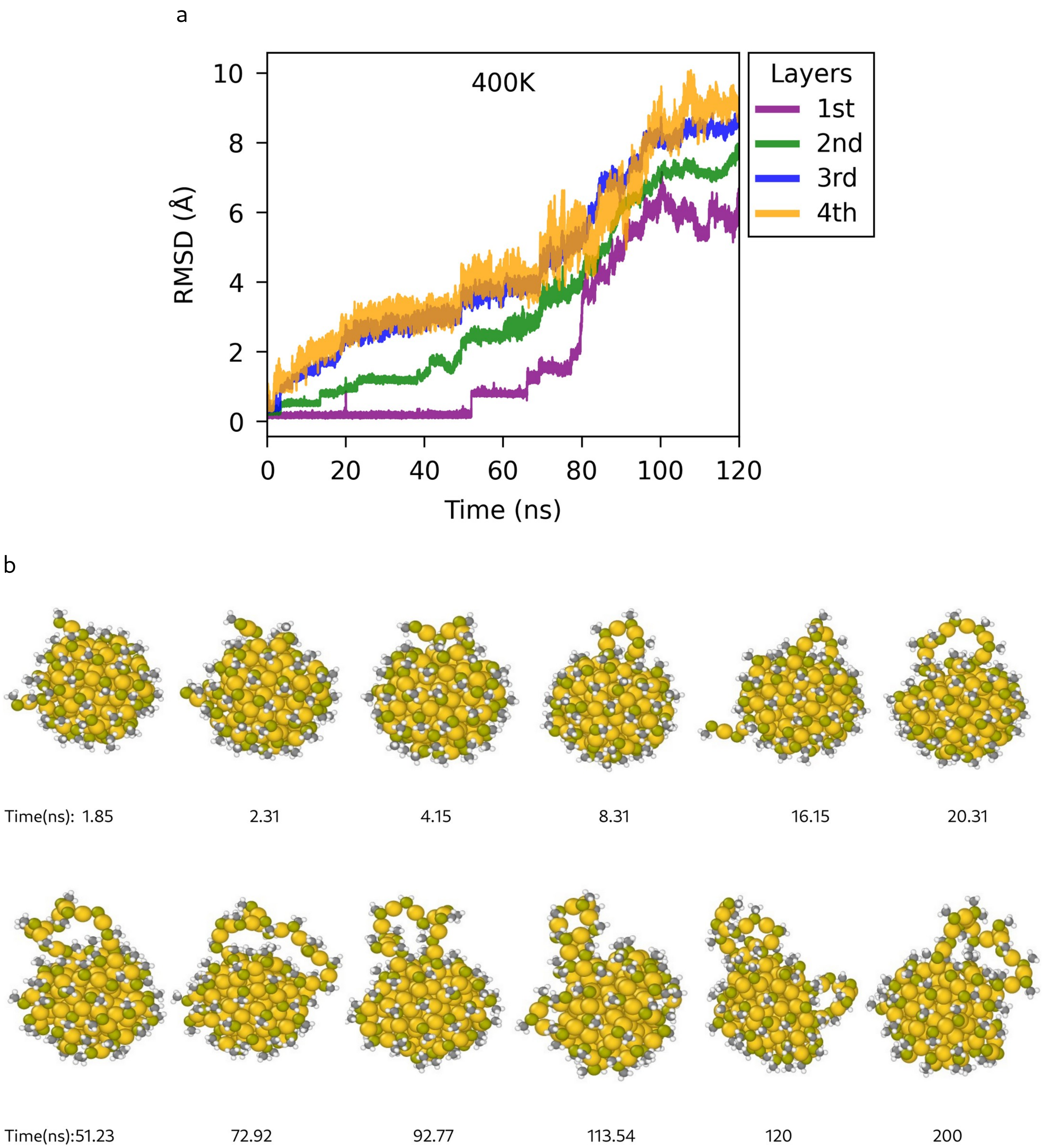

Supplementary Figure 19. Layer-resolved RMSD of $Au_{144}(SCH_3)_{60}$ at 400 K from the first MD replica (I). a RMSD of all atoms in $Au_{144}(SR)_{60}$ during MD simulation over 120 ns at **400 K (I)**. Layers are shown in purple (1st), green (2nd), blue (3rd), and orange (4th/in ligand shell). **b** Selected snapshots illustrating the atomic evolution at different time points (ns) during the MD simulation.

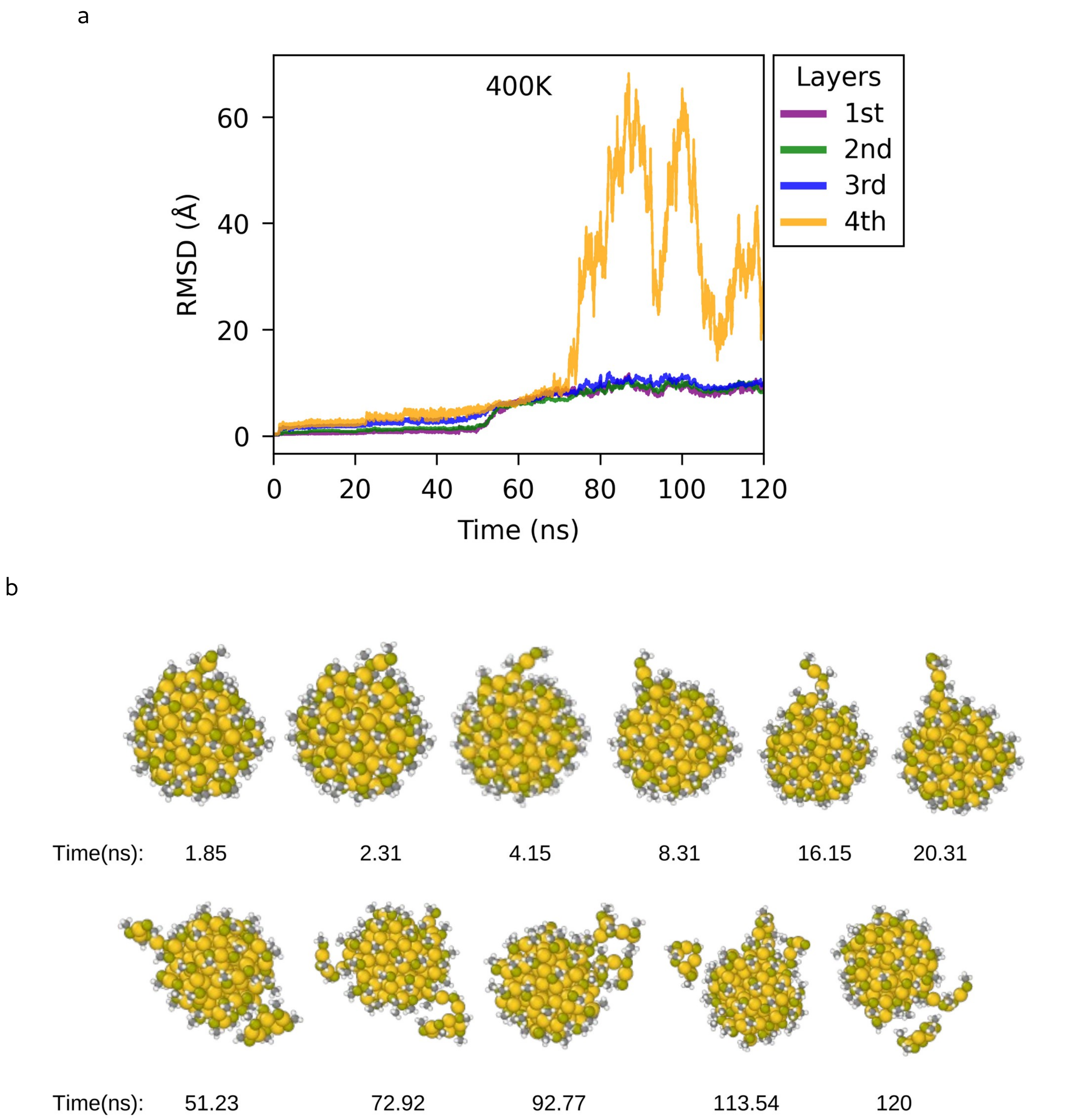

Supplementary Figure 20. Layer-resolved RMSD of $Au_{144}(SCH_3)_{60}$ at 400 K from the second MD replica (II). a RMSD of all atoms in $Au_{144}(SR)_{60}$ during MD simulation at **400 K (II)**. The RMSD plot tracks structural fluctuations over 120 ns. Layers are shown in purple (1st), green (2nd), blue (3rd), and orange (4th/in ligand shell). **b** Selected snapshots illustrating the atomic evolution at different time points (ns) during the MD simulation.

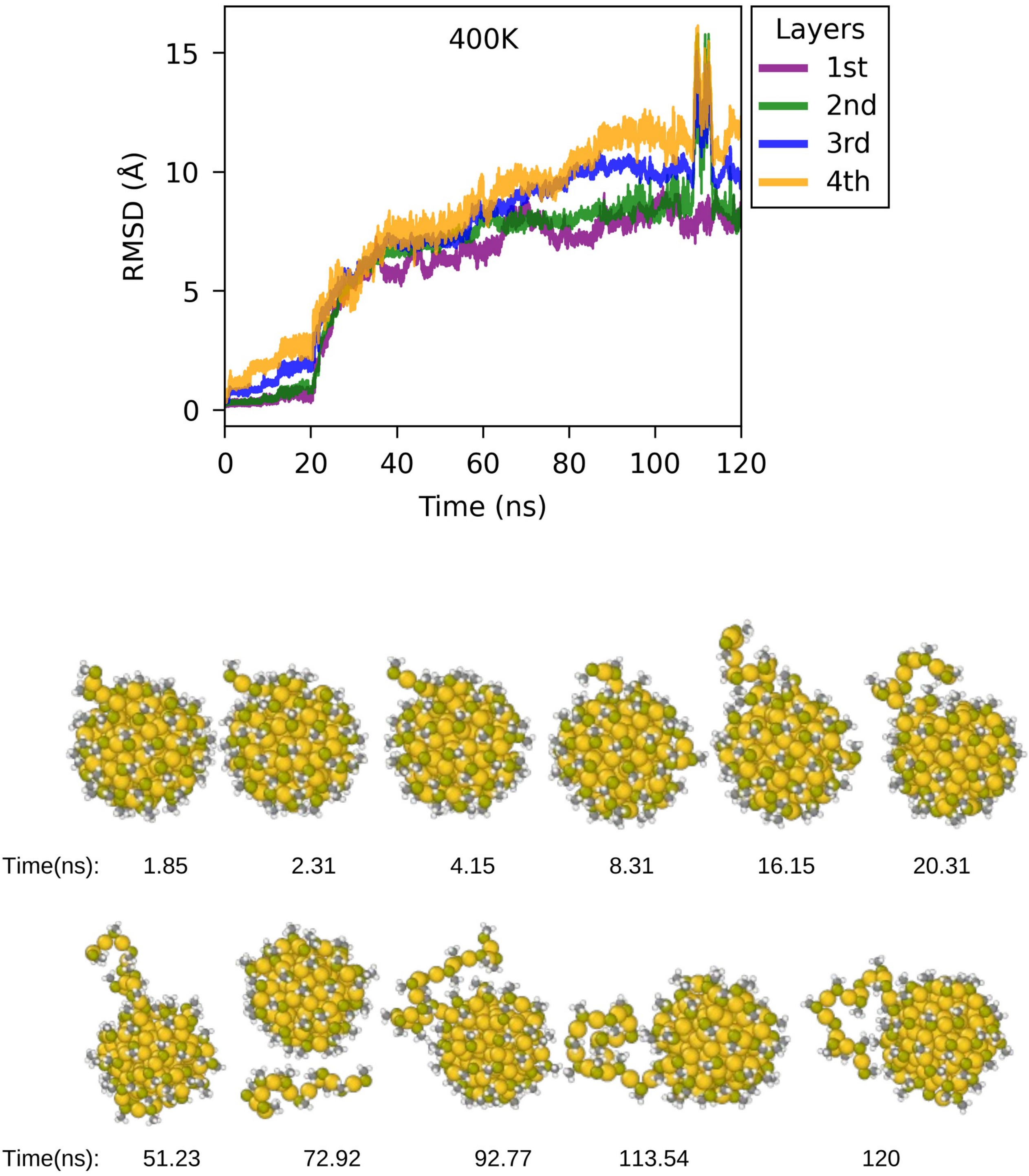

Supplementary Figure 21. Layer-resolved RMSD of $Au_{144}(SCH_3)_{60}$ at 400 K from the third MD replica (III). a RMSD of all atoms in $Au_{144}(SR)_{60}$ during MD simulation at **400 K (III)**. The RMSD plot tracks structural fluctuations over 120 ns. Layers are shown in purple (1st), green (2nd), blue (3rd), and orange (4th/in ligand shell). **b** Selected snapshots illustrating the atomic evolution at different time points (ns) during the MD simulation.

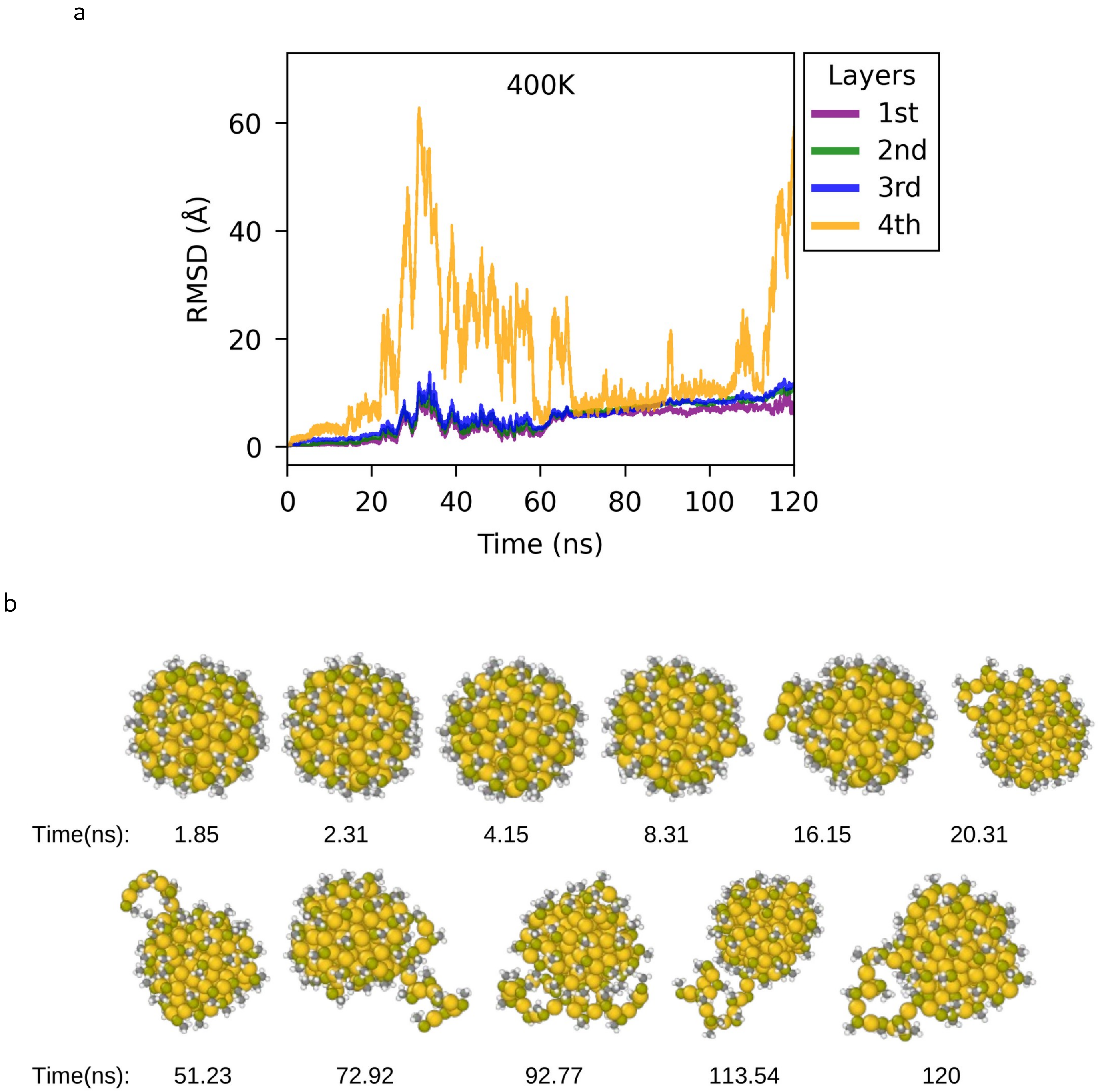

Supplementary Figure 22. Layer-resolved RMSD of $Au_{144}(SCH_3)_{60}$ at 400 K from the fourth MD replica (IV). a RMSD of all atoms in $Au_{144}(SR)_{60}$ during MD simulation at **400 K (IV)**. The RMSD plot tracks structural fluctuations over 120 ns. Layers are shown in purple (1st), green (2nd), blue (3rd), and orange (4th/in ligand shell). **b** Selected snapshots illustrating the atomic evolution at different time points (ns) during the MD simulation.

a

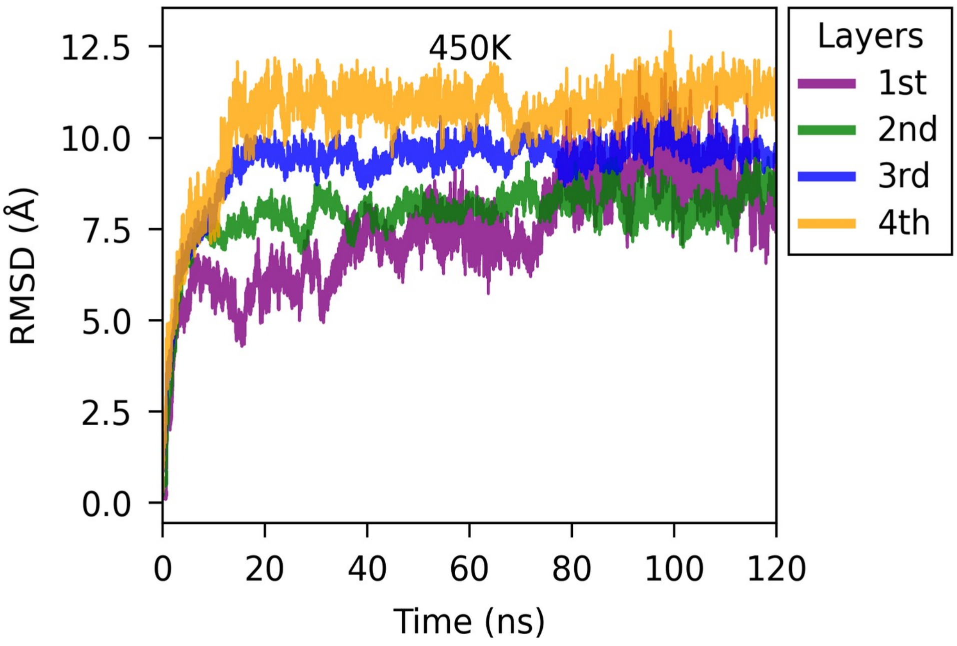

b

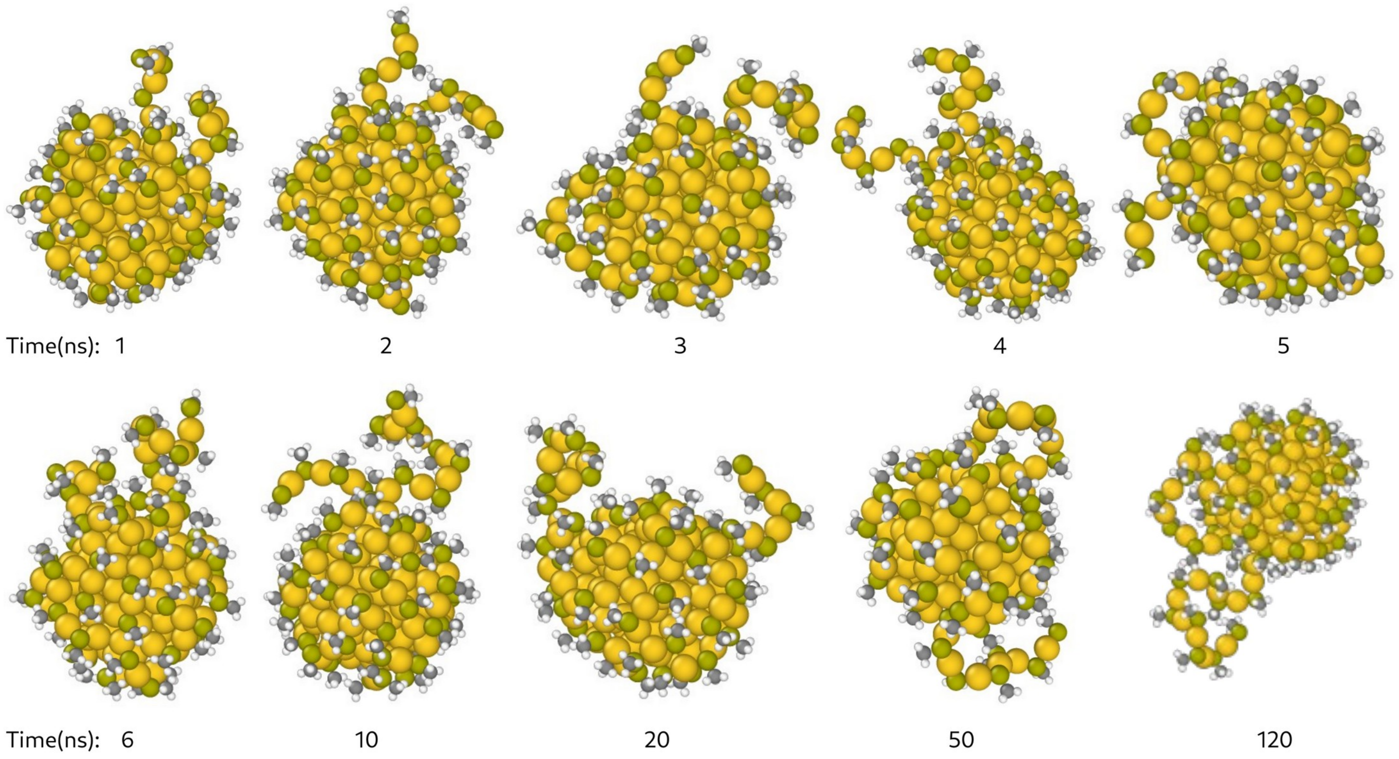

Supplementary Figure 23. Layer-resolved RMSD of $Au_{144}(SCH_3)_{60}$ at 450 K from the first MD replica (I). a RMSD of all atoms in $Au_{144}(SR)_{60}$ during MD simulation over 120 ns at **450 K (I)**. Layers are shown in purple (1st), green (2nd), blue (3rd), and orange (4th/in ligand shell). **b** Selected snapshots illustrating the atomic evolution at different time points (ns) during the MD simulation.

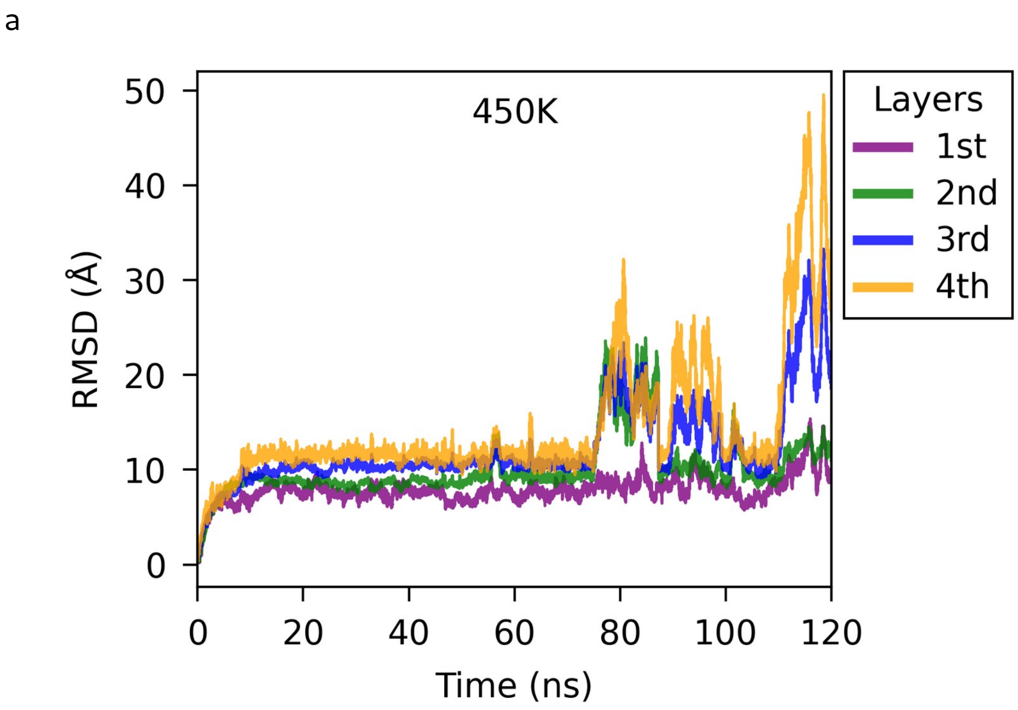

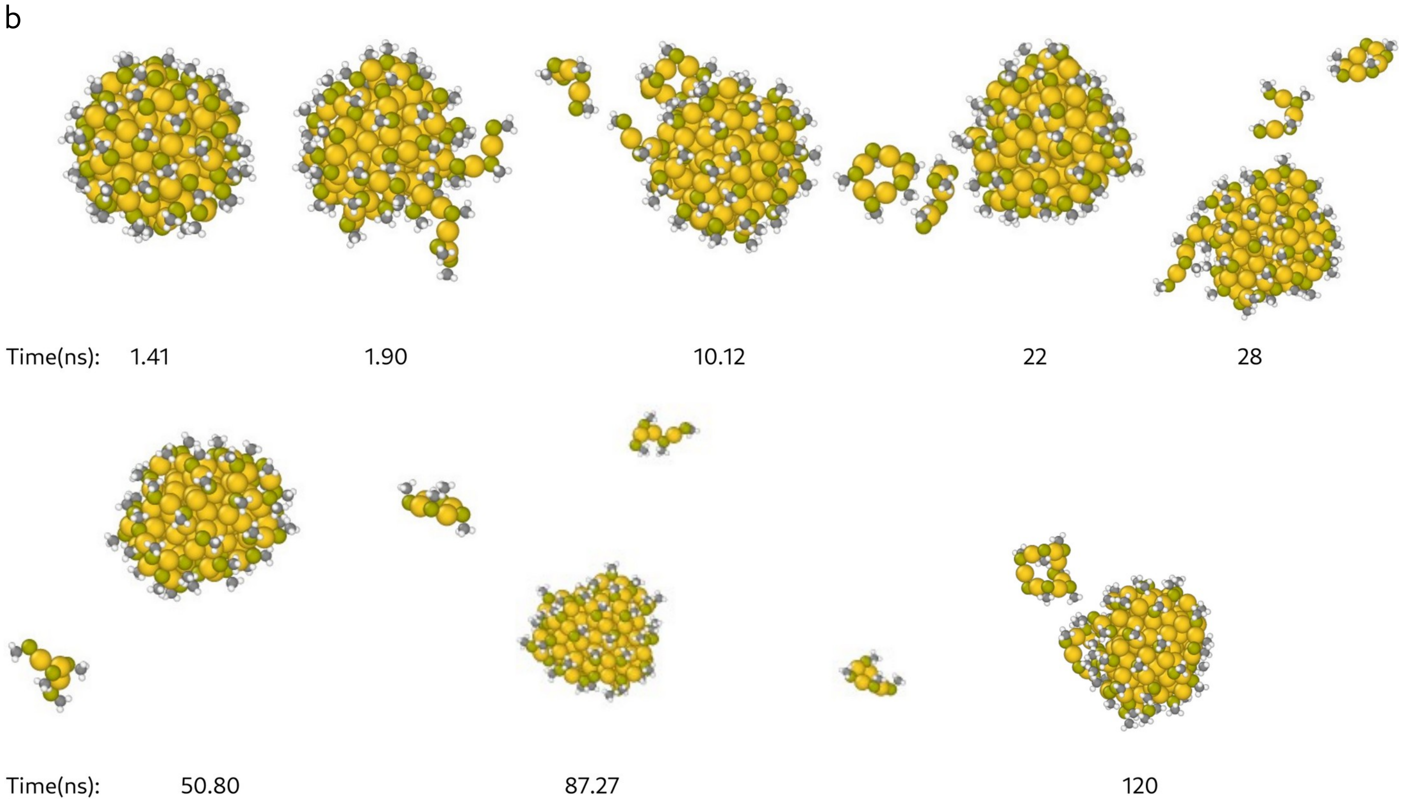

Supplementary Figure 24. Layer-resolved RMSD of $Au_{144}(SCH_3)_{60}$ at 450 K from the second MD replica (II). a RMSD of all atoms in $Au_{144}(SR)_{60}$ during MD simulation over 87.27 ns at **450 K (II)**. Layers are shown in purple (1st), green (2nd), blue (3rd), and orange (4th/in ligand shell). **b** Selected snapshots illustrating the atomic evolution at different time points (ns) during the MD simulation.

a

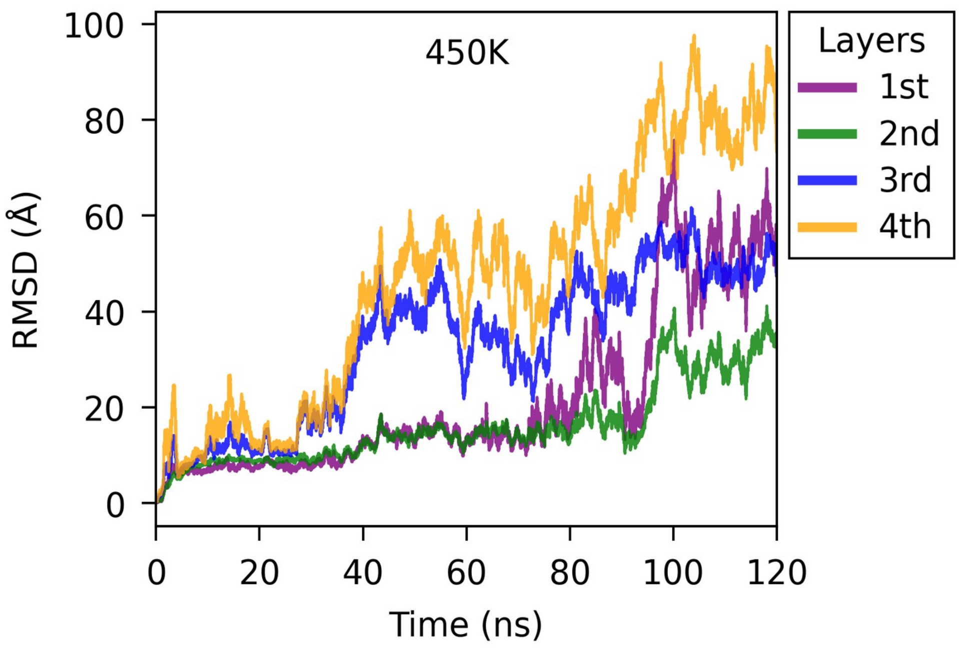

b

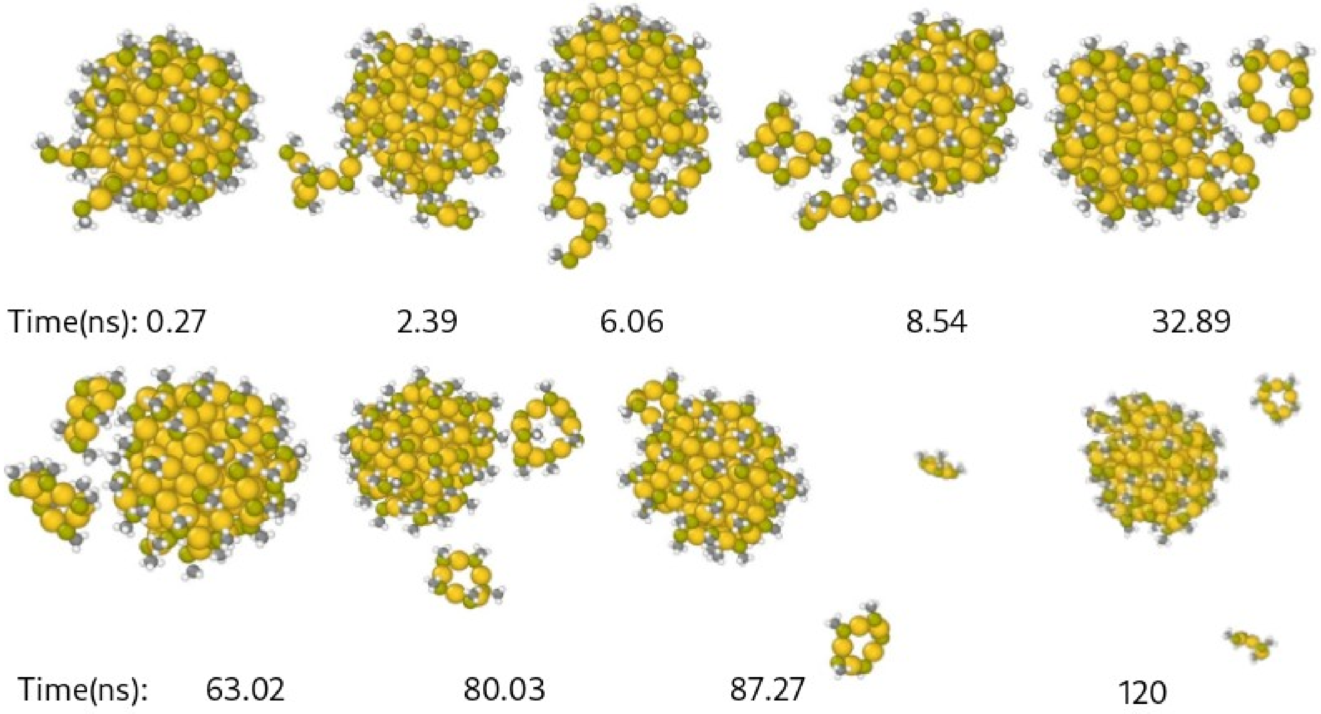

Supplementary Figure 25. Layer-resolved RMSD of $Au_{144}(SCH_3)_{60}$ at 450 K from the third MD replica (III). a RMSD of all atoms in $Au_{144}(SR)_{60}$ during MD simulation over 89.85 ns at **450 K (III)**. Layers are shown in purple (1st), green (2nd), blue (3rd), and orange (4th/in ligand shell). **b** selected snapshots illustrating the atomic evolution at different time points (ns) during the MD simulation.

a

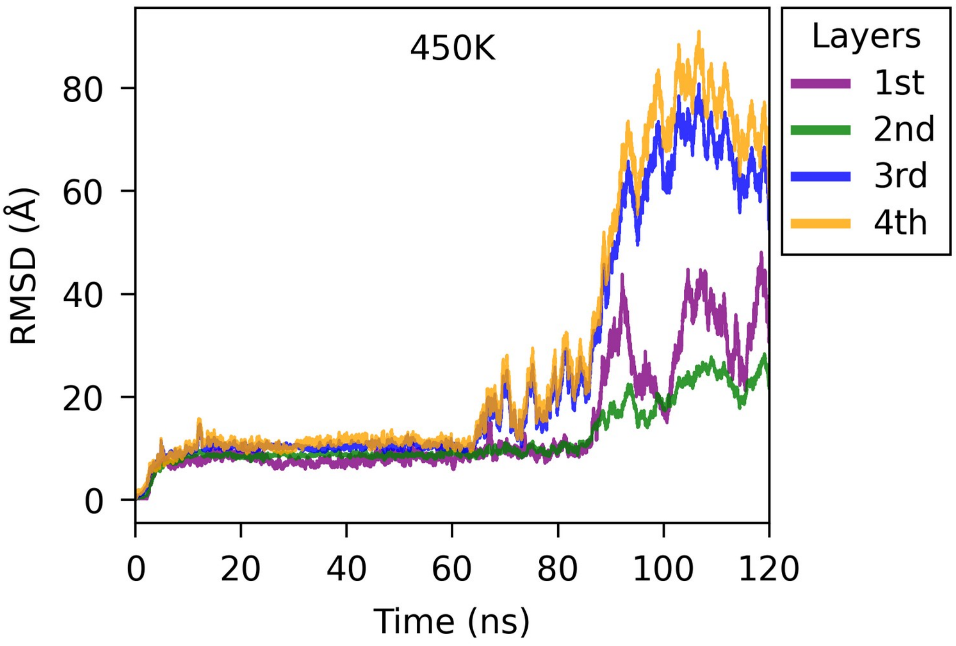

b

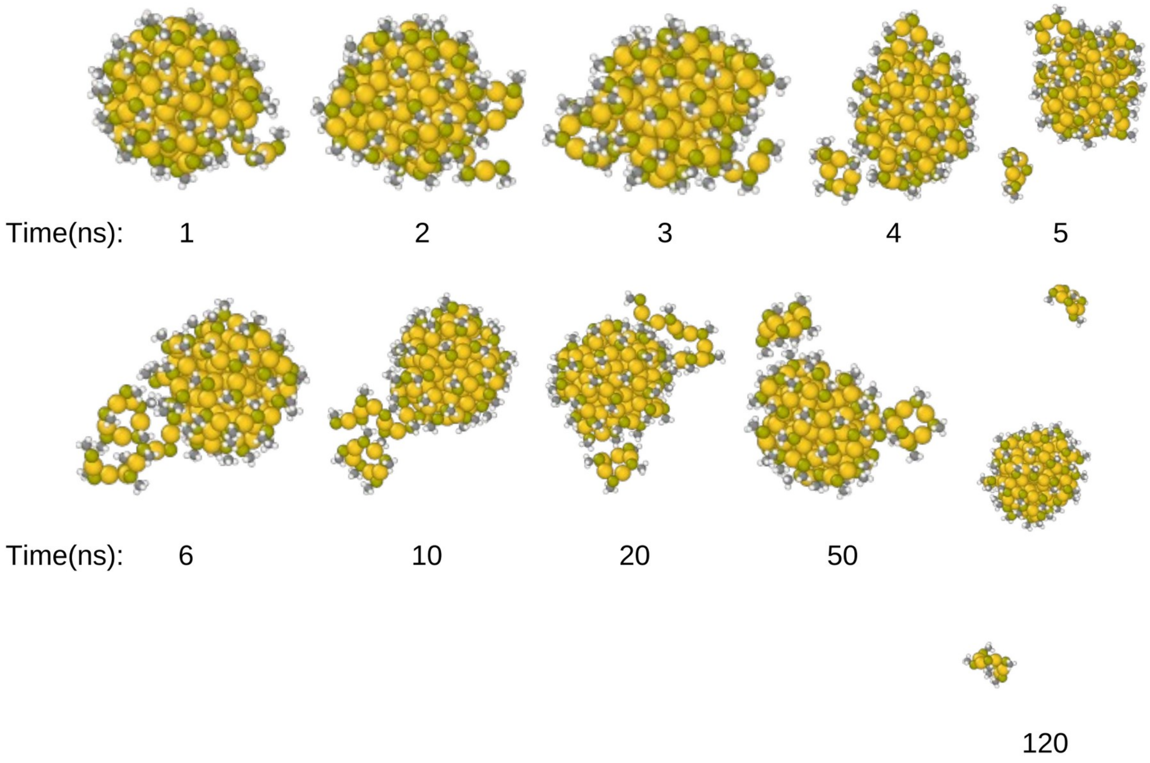

Supplementary Figure 26. Layer-resolved RMSD of $Au_{144}(SCH_3)_{60}$ at 450 K from the fourth MD replica (IV). a RMSD of all atoms in $Au_{144}(SR)_{60}$ during MD simulation at **450 K (IV)**. The RMSD plot tracks structural fluctuations over 120 ns. Layers are shown in purple (1st), green (2nd), blue (3rd), and orange (4th/in ligand shell). **b** Selected snapshots illustrating the atomic evolution at different time points (ns) during the MD simulation.

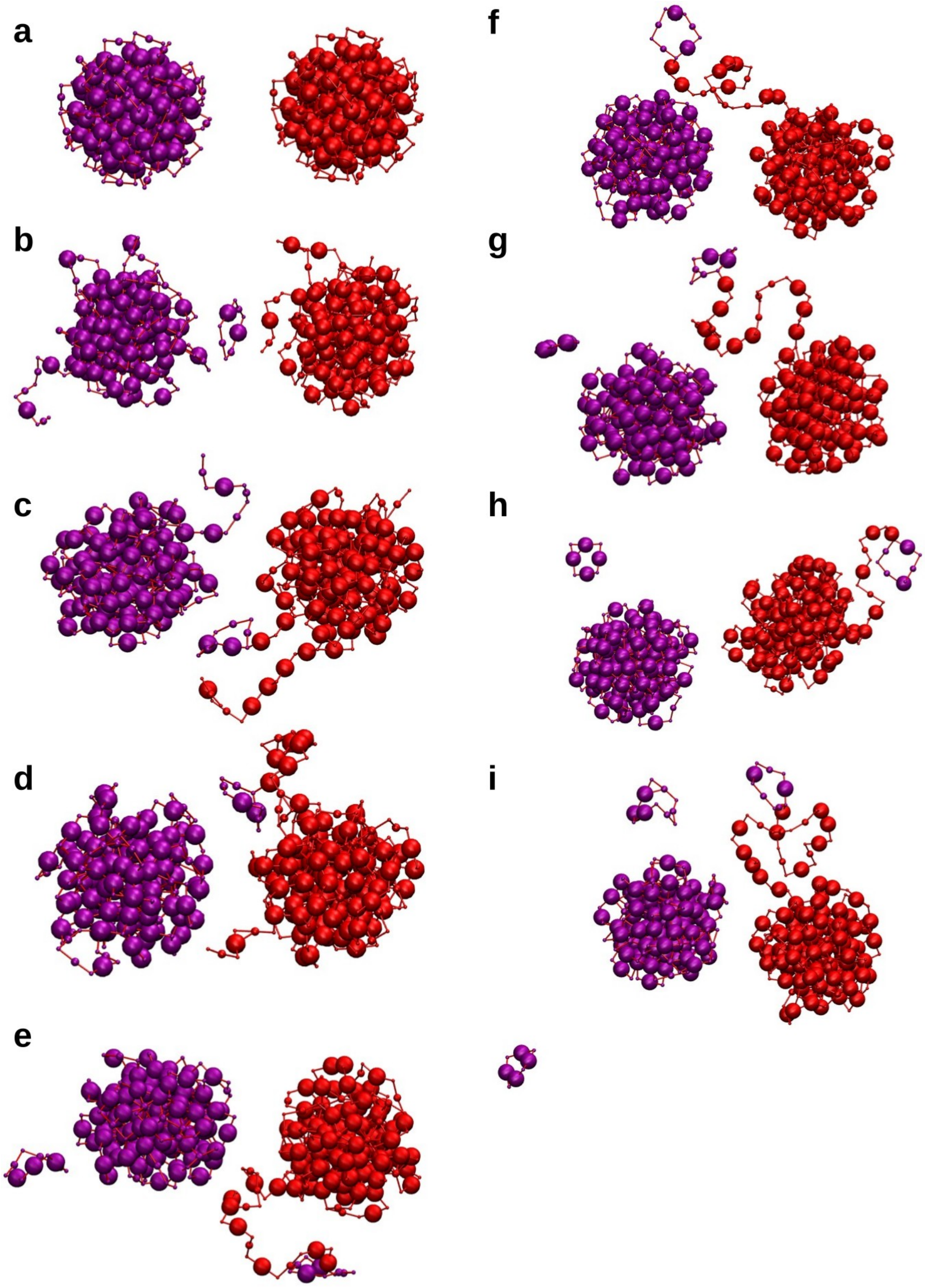

Supplementary Figure 27. Fusion dynamics of two Au_{144} $(SCH_3)_{60}$ at 500K. Selected snapshots shown in panels (**a-i**) from MD simulations of two Au_{144} $(SCH_3)_{60}$ clusters at **500 K** positioned **3.08 Å** apart (measured as the shortest distance between the outermost H atoms of each cluster), shown in red and purple for distinction. The snapshots are taken at: **a**: 0 ns, **b**: 1.272 ns, **c**: 5.341 ns, **d**: 12.650 ns, **e**: 13.915 ns, **f**: 37.107 ns, **g**: 43.103 ns, **h**: 46.806 ns, **i**: 50.428 ns. Core Au atoms are represented as the largest spheres, Au atoms in ligand staples are of intermediate size, and sulfur atoms are the smallest.

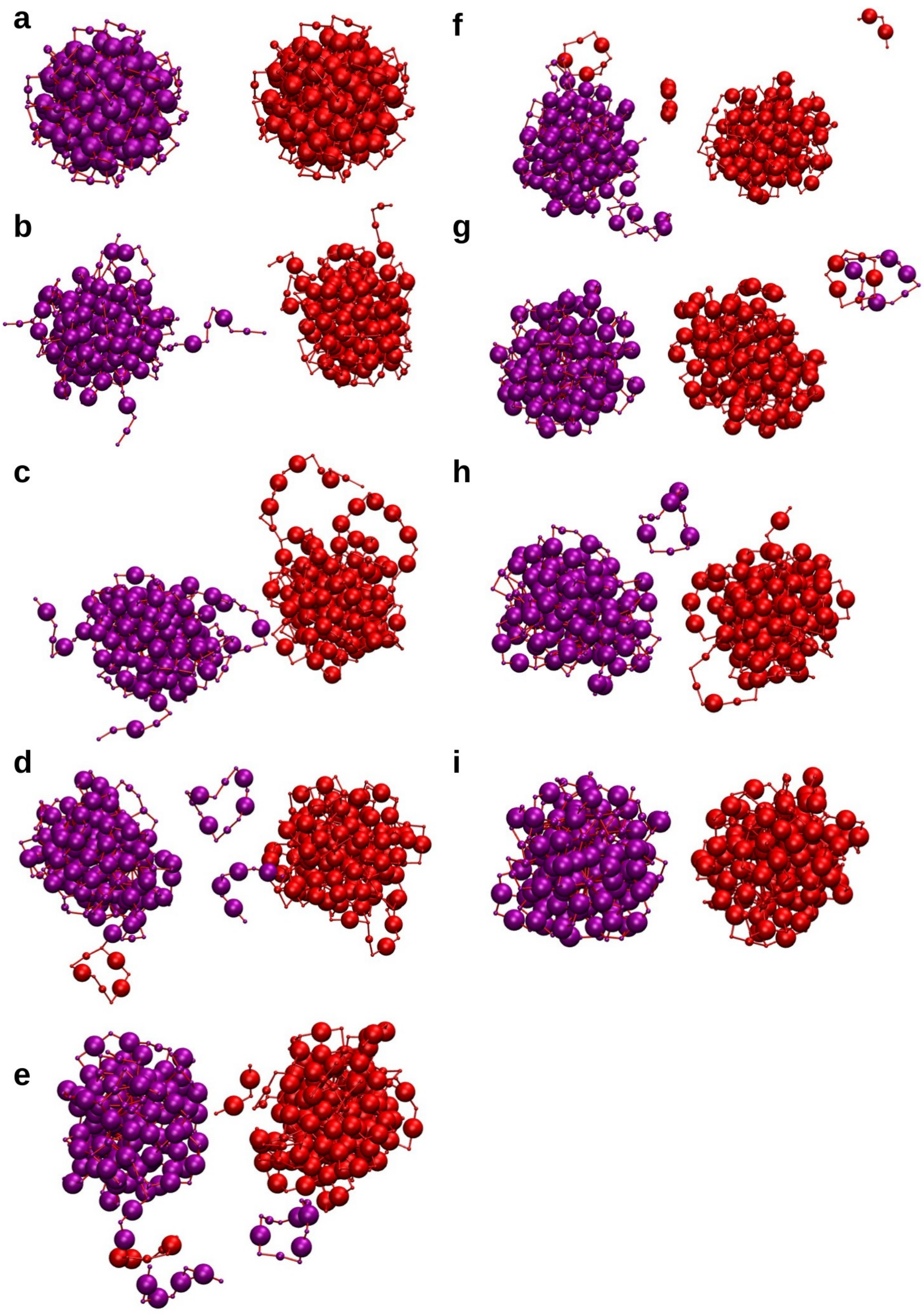

Supplementary Figure 28. Fusion dynamics of two Au_{144} $(SCH_3)_{60}$ at 550K. Selected snapshots shown in panels (a-i) from MD simulations of two Au_{144} $(SCH_3)_{60}$ clusters at **550 K** positioned **3.08 Å** apart (measured as the shortest distance between the outermost H atoms of each cluster), shown in red and purple for distinction. The snapshots are taken at: **a**: 0 ns, **b**: 0.52 ns, **c**: 2.42 ns, **d**: 12.65 ns, **e**: 17.28 ns, **f**: 23.31 ns, **g**: 35.01 ns, **h**: 41.91 ns, **i**: 48.85 ns. Core Au atoms are represented as the largest spheres, Au atoms in ligand staples are of intermediate size, and sulfur atoms are the smallest. The methyl groups were excluded for clarity.

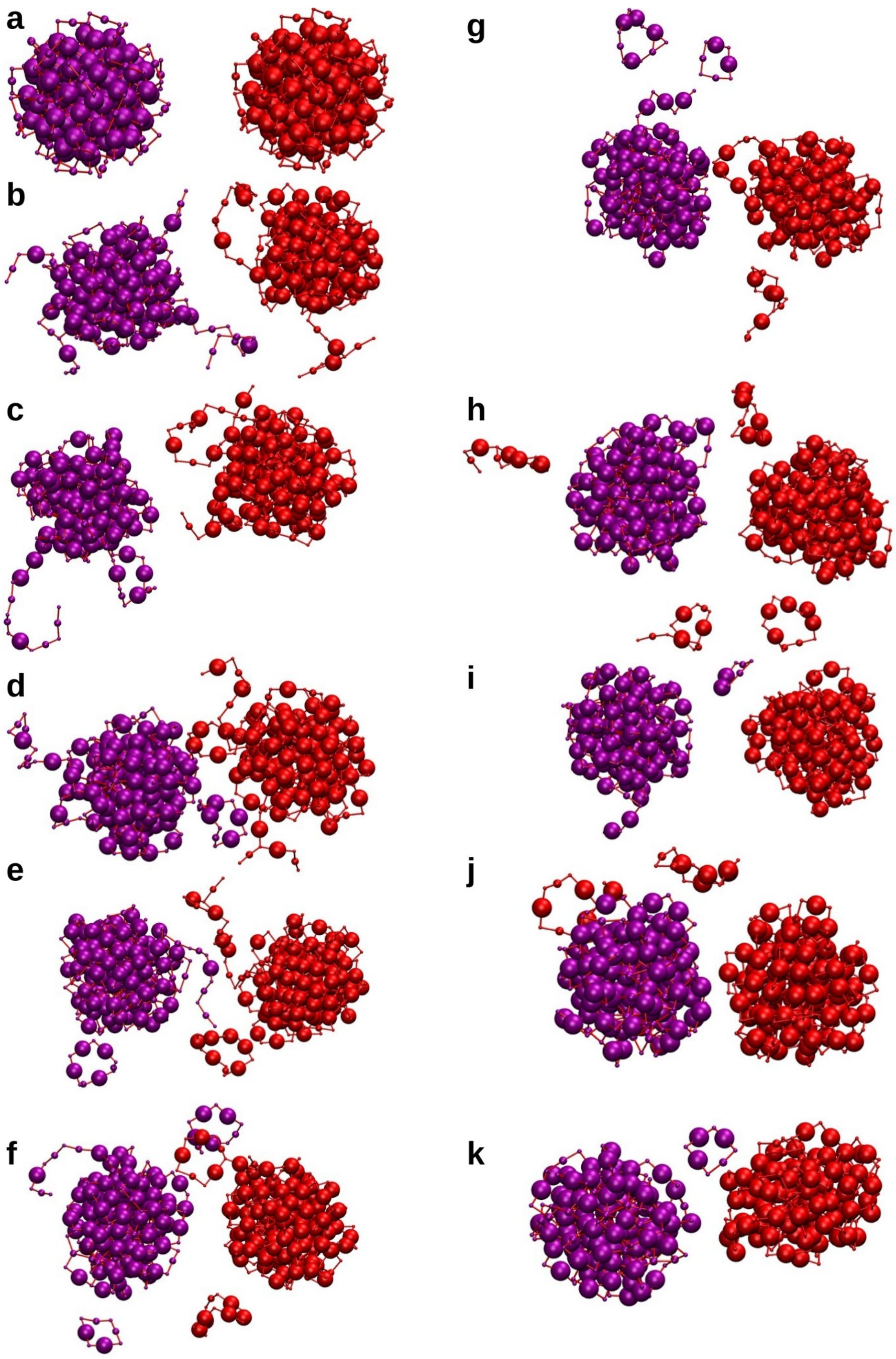

Supplementary Figure 29. Fusion dynamics of two Au_{144} $(SCH_3)_{60}$ at 500K. Selected snapshots shown in panels (a-k) from MD simulations of two Au_{144} $(SCH_3)_{60}$ clusters at **500 K** positioned **1.6 Å** apart (measured as the shortest distance between the outermost H atoms of each cluster), shown in red and purple for distinction. The snapshots are taken at: **a**: 0 ns, **b**: 1.539 ns, **c**: 6.016 ns, **d**: 7.974 ns, **e**: 15.949 ns, **f**: 20.846 ns, **g**: 28.960 ns, **h**: 43.510 ns, **i** 45.05 , **j** 52.18, **k** 68.71. Core Au atoms are represented as the largest spheres, Au atoms in ligand staples are of intermediate size, and sulfur atoms are the smallest. The methyl groups were excluded for clarity.

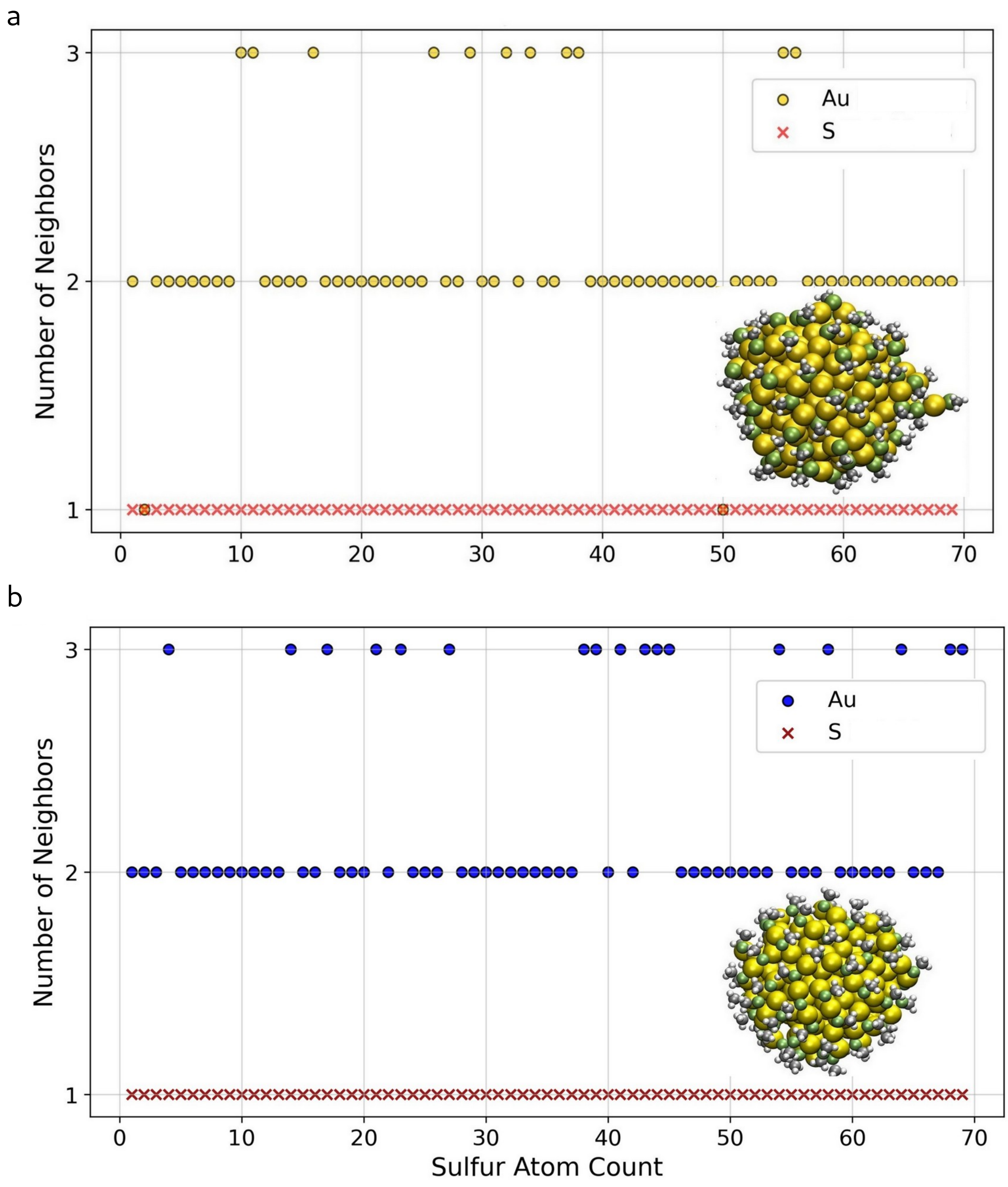

Supplementary Figure 30. Coordination environment of S atoms in $Au_{239}(SR)_{69}$. Number of Au and S neighbors for each S atom: (**a**) from the final MD snapshot of two $Au_{144}(SR)_{60}$ clusters at **550 K** (Au neighbors: yellow circles; S neighbors: red crosses), and (**b**) after cooling to **300 K** and geometry optimization using the ACE potential (Au neighbors: blue circles; S neighbors: red crosses). Insets show the two $Au_{239}(SR)_{69}$ structures with Au (yellow), S (green), C (gray), and H (white) spheres.

Supplementary Tables

Supplementary Tables 1. Temperature-dependent fragmentation of two $Au_{144}(SCH_3)_{60}$ clusters. Summary of fragmentation events observed in MD simulations of two $Au_{144}(SCH_3)_{60}$ clusters at **400 K**, **500 K**, and **550 K**. Simulations were performed at two initial cluster-cluster separations: **3.08 Å** (for **400 K**, **500 K**, and **550 K**) and **1.6 Å** (for **500 K** and **550 K**).

T (K)/ d (Å)	Fragment molecular formula
400/ 3.08	$Au_4(SR)_4$<br>$Au_7(SR)_7$
500/ 3.08	$Au_4(SR)_4$<br>$Au_6(SR)_6$<br>$Au_{15}(SR)_{15}$
550/ 3.08	$Au(SR)_2$<br>$Au_4(SR)_5$<br>$Au_5(SR)_5$<br>$Au_7(SR)_7$
500/ 1.6	$Au_4(SR)_4$<br>$Au_5(SR)_5$<br>$Au_6(SR)_6$<br>$Au_7(SR)_7$
550/ 1.6	$Au(SR)_2$<br>$Au_3(SR)_4$<br>$Au_4(SR)_4$<br>$Au_5(SR)_5$<br>$Au_6(SR)_6$<br>$Au_8(SR)_8$
