# Supplementary material for: Thermal dynamics and coalescence of Au144(SR)60 clusters from a machine-learned potential
Source: Nat Commun. 2025 Dec 18;17:971. doi: 10.1038/s41467-025-67700-w (PMC12847723; doi:10.1038/s41467-025-67700-w)
Supplement: Supplementary file 4 — Source Data [file 41467_2025_67700_MOESM4_ESM.zip › source data/Figure_3.zip!/Figure_3/Figure3.pdf]

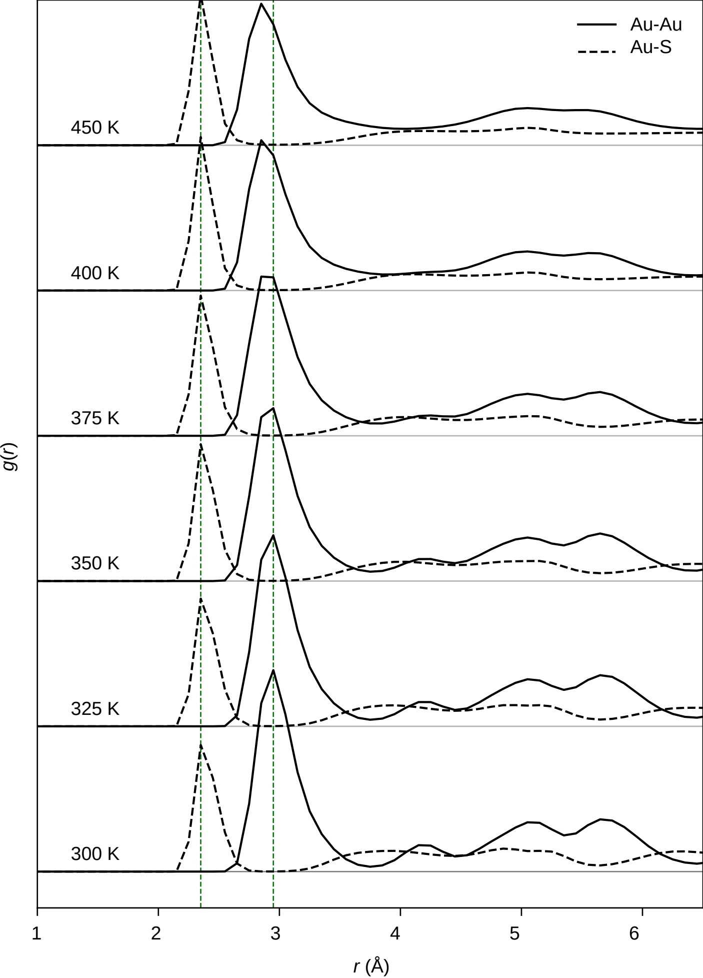

Au-Au
Au-S
450 K
400 K
375 K
350 K
325 K
300 K
g(r)
r (Å)
1
2
3
4
5
6
