# Supplementary material for: Thermal dynamics and coalescence of Au144(SR)60 clusters from a machine-learned potential
Source: Nat Commun. 2025 Dec 18;17:971. doi: 10.1038/s41467-025-67700-w (PMC12847723; doi:10.1038/s41467-025-67700-w)
Supplement: Supplementary file 4 — Source Data [file 41467_2025_67700_MOESM4_ESM.zip › source data/Figure_4.zip!/Figure_4/plot_Figure4/Figure4.pdf]

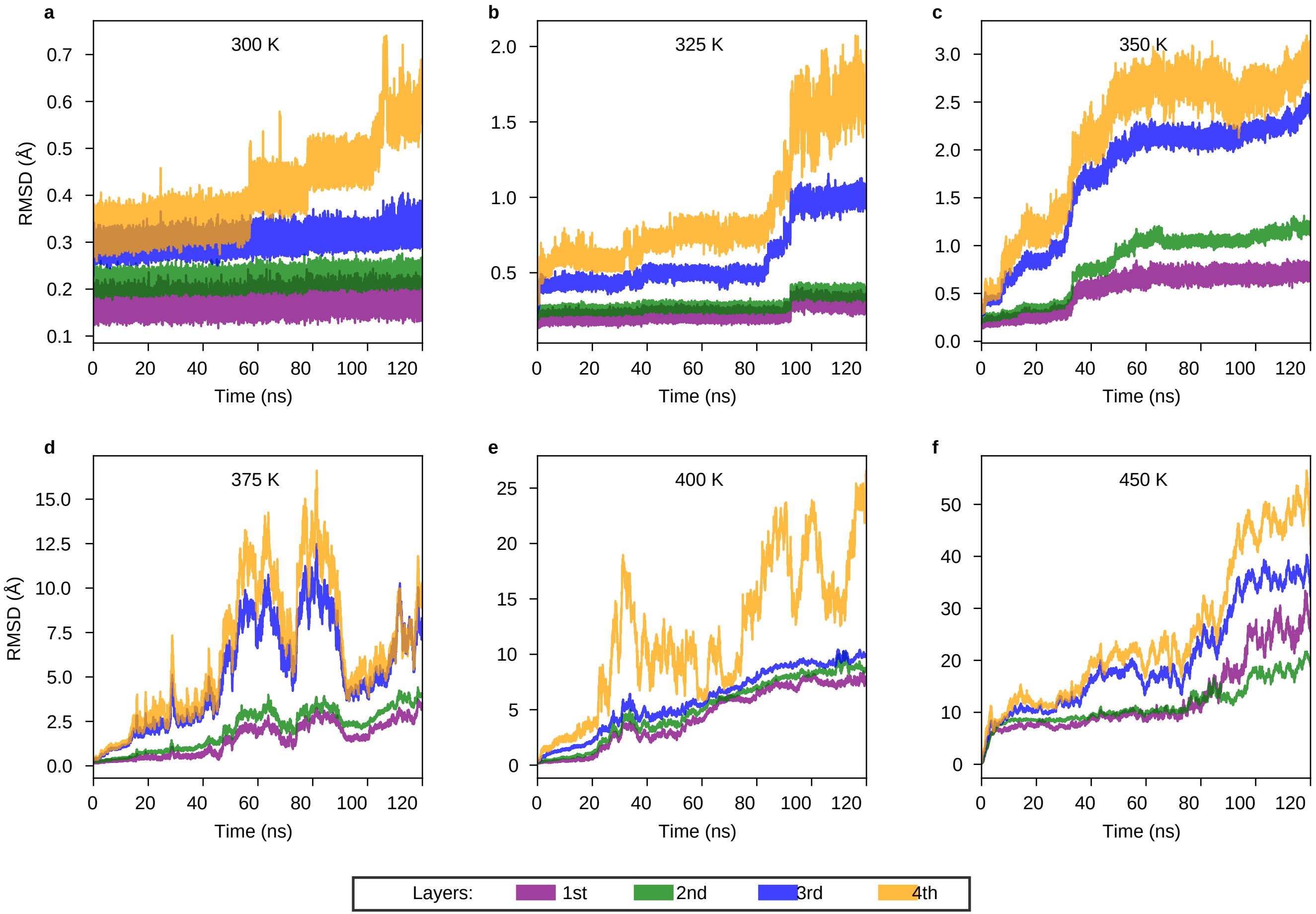

a
300 K
b
325 K
c
350 K
d
375 K
e
400 K
f
450 K
RMSD (Å)
Time (ns)
Layers:
1st
2nd
3rd
4th
