# Supplementary material for: Thermal dynamics and coalescence of Au144(SR)60 clusters from a machine-learned potential
Source: Nat Commun. 2025 Dec 18;17:971. doi: 10.1038/s41467-025-67700-w (PMC12847723; doi:10.1038/s41467-025-67700-w)
Supplement: Supplementary file 4 — Source Data [file 41467_2025_67700_MOESM4_ESM.zip › source data/Figure_4.zip!/Figure_4/375/mean_RMSD/mean_rmsd_375K.pdf]

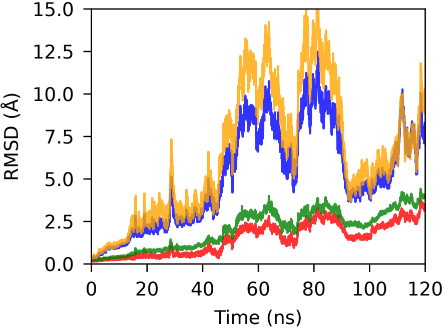

RMSD (Å)
15.0
12.5
10.0
7.5
5.0
2.5
0.0
0
20
40
60
80
100
120
Time (ns)
